# Motor training improves coordination and anxiety in symptomatic *Mecp2*-null mice despite impaired functional connectivity within the motor circuit

**DOI:** 10.1126/sciadv.abf7467

**Published:** 2021-10-22

**Authors:** Yuanlei Yue, Pan Xu, Zhichao Liu, Xiaoqian Sun, Juntao Su, Hongfei Du, Lingling Chen, Ryan T. Ash, Stelios Smirnakis, Rahul Simha, Linda Kusner, Chen Zeng, Hui Lu

**Affiliations:** 1 Department of Pharmacology and Physiology, School of Medicine and Health Sciences, The George Washington University, Washington, DC 20037, USA.; 2 Department of Physics, Columbian College of Arts and Sciences, The George Washington University, Washington, DC 20037, USA.; 3 Department of Computer Science, School of Engineering and Applied Science, The George Washington University, Washington, DC 20037, USA.; 4 Department of Statistics, Columbian College of Arts and Sciences, The George Washington University, Washington, DC 20037, USA.; 5 Department of Psychiatry, Stanford University, Palo Alto, CA 94305, USA.; 6 Department of Neurology, Brigham and Women’s Hospital, Jamaica Plain VA Hospital, Harvard Medical School, Boston, MA 02115, USA.

## Abstract

Rett syndrome (RTT) is a severe neurodevelopmental disorder caused by loss of function of the X-linked methyl-CpG–binding protein 2 (*MECP2*). Several case studies report that gross motor function can be improved in children with RTT through treadmill walking, but whether the MeCP2-deficient motor circuit can support actual motor learning remains unclear. We used two-photon calcium imaging to simultaneously observe layer (L) 2/3 and L5a excitatory neuronal activity in the motor cortex (M1) while mice adapted to changing speeds on a computerized running wheel. Despite circuit hypoactivity and weakened functional connectivity across L2/3 and L5a, the *Mecp2*-null circuit’s firing pattern evolved with improved performance over 2 weeks. Moreover, trained mice became less anxious and lived 20% longer than untrained mice. Because motor deficits and anxiety are core symptoms of RTT, which is not diagnosed until well after symptom onset, these results underscore the benefit of motor learning.

## Teaser

Even symptomatic *Mecp2*-null mice can develop motor skills, which improve behavior and extend life span by 20%.

## INTRODUCTION

Experience tells us that we develop a skill by repeated efforts at a particular outcome, whether the task is cognitive, physical, or both. Even a relatively simple task such as walking involves a combination of both hardwired abilities and plasticity for adapting basic movements to different environmental demands while achieving reliable outcomes. The development of motor skills relies heavily on the motor cortex (M1), particularly layers 2/3 and 5 (L2/3 and L5), which undergoes reorganization during learning (*1*–*5*). Motor learning enhances synaptic connections in M1 by coordinating the clustering of dendritic spines, forming the structural basis for storing motor memory (*6*, *7*). L2/3 and L5a provide excitatory input to the major motor output layer, L5b (*8*). The microcircuits in L2/3 integrate sensory feedback, motor planning, and primitives into motor output, whereas L5a neurons play crucial roles in action selection, motor control, sequence learning, and habit formation (*3*, *9*). Neurons in these two layers are reciprocally connected, but we are only beginning to learn how they encode motor skills.

We were particularly interested in understanding how the function of the M1 circuit is altered in a neurodevelopmental disorder known as Rett syndrome (RTT), which is caused by mutations in the X-linked methyl-CpG–binding protein 2 (*MECP2*) (*10*) and appears to involve a loss of the ability to learn. RTT is notable for the unusual course of the disease: Children (typically girls) develop normally until about 18 to 24 months of age, when they undergo a period of regression wherein they lose acquired motor, cognitive, and social milestones and do not make further gains (*11*). They stop speaking, they develop hand stereotypies that replace purposeful hand use, they develop apraxia and ataxia if they remain ambulatory, and they develop a host of additional neurological symptoms such as seizures, respiratory dysrhythmias, and anxiety. There is no neurodegeneration; mice in which *Mecp2* is reexpressed in adulthood are completely rescued (*12*). This indicates that there is no fundamental structural abnormality, although *Mecp2*-deficient neurons in mice are smaller and are more closely packed than in wild-type (WT) animals (*13*, *14*). MeCP2 expression rises postnatally and seems to have roles in maintaining the physiology of mature neurons (*15*–*17*), but the *Mecp2-*heterozygous hippocampal circuit was still able to be activated by deep brain stimulation (DBS) to a sufficient degree to rescue learning and memory deficit (*18*) and reduce the abnormal hypersynchrony of neuronal activity (*19*). Considering that the motor cortex also remains structurally intact during regression, we wondered whether the motor circuit was, despite evidence to the contrary, still capable of learning after regression has set in and specifically whether the motor cortical circuit would respond to a less directed source of stimulation, namely, exercise.

To study M1 activity and particularly cross-layer interaction during motor skill acquisition in WT and *Mecp2*-null mice, we monitored L2/3 and L5a of M1 in the mice using two-photon calcium imaging during motor training. The question was what kind of motor learning task would be suitable. Early symptomatic *Mecp2*-null mice are considerably weaker, less coordinated, and less active than WT mice (*20*), so voluntary exercise would not work. On the other hand, classic motor learning tasks in rodents, such as operating a lever to get food pellets, depend on operant conditioning, i.e., pairing a specific behavior with a reward. The involvement of reward circuits might therefore complicate experimental investigation of responses in the motor cortex (*21*), as RTT is known to involve abnormalities in mesocorticolimbic reward pathways (*22*). We therefore developed a forced exercise paradigm, where the mice are placed on a computerized running wheel whose speed can be controlled by the experimenter; the skill to be learned is to adapt to changing speeds across multiple training sessions. This experimental paradigm does not require food reward or water restriction, and, because locomotion is partly hardwired, it requires minimal involvement of the higher-order motor system. While mice underwent 2 weeks of training on the computerized wheel, we conducted two-photon calcium imaging to record neurons in L2/3 and L5.

We found that training for 2 weeks reshaped M1 circuit dynamics in conjunction with improvement in motor skill on the wheel. Our evidence supports a model in which motor learning strengthens the connectivity of a subgroup of neurons for storing information while decreasing connectivity among the rest of the population. We also show that despite reduced cross-layer connectivity, ~20% overall lower event rate, and impairment in maintaining functional connectivity more than a couple of days, the *Mecp2*-null motor cortex retains sufficient plasticity to support some motor learning. More unexpectedly, a mere 2 weeks of training was able to improve motor coordination on different assays (ladder walking and accelerating rotarod tests), reduce anxiety (in the open field and elevated plus maze tests), and extend the life span of *Mecp2*-null mice by 20%.

## RESULTS

### Male *Mecp2*-null mice are impaired in wheel running but can improve

**Fig. 1.**
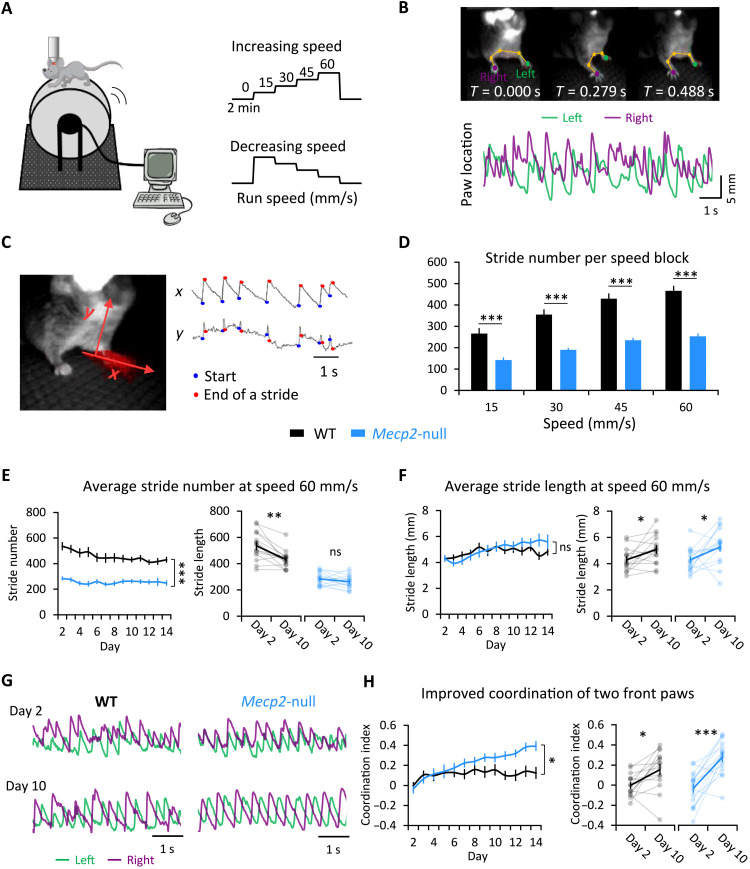
Following the development of motor skill in forced wheel running. (**A**) The experimental setup. A mouse, whose head is fixed under a two-photon microscope, runs on the computerized wheel, whose speed is controlled by the experimenter. Scale bar, 50 μm. (**B**) Top: Video frames of forepaw movements at three representative time points, with paw locations labeled by DeepLabCut. Bottom: Sample temporal traces for the left and right paws of one mouse moving at 60 mm/s on the wheel. The paw location at any given moment is calculated as the distance between the median paw location and location at that time. (**C**) Automated stride analysis. *x* and *y* axes represent horizontal and vertical paw movements, respectively. A step is represented by the rising phase of a spike in *x* and a complete spike in *y*. (**D**) The average number of strides completed by the left paw at each speed (during increasing-speed mode) over the training period. Error bars represent means ± SE. *n* = 13 mice for WT group; *n* = 15 for *Mecp2*-null group. **P < 0.01, repeated measures analysis of variance (RM-ANOVA) with Sidak’s post hoc test. (**E**) Stride number (left) and stride length (right) at speed 60 mm/s over 14 successive days. **P < 0.01 and ***P < 0.001. (**F**) Left: Comparison of stride length between genotypes every 10 s during the 2-min block of speed 60 mm/s on days 2 and 10, fitted with a straight line. Right: Within-genotype comparisons of stride length during early learning (day 2) and consolidation (day 10) phases. *P < 0.05. (**G**) Sample temporal traces for the forepaws of a mouse running at speed 60 mm/s shows improved coordination (anticorrelation) by day 10. (**H**) Coordination at speed 60 mm/s improves over the 14-day training period. The coordination index was calculated as the negative value of the correlation coefficient of the two forepaw traces during the 2-min block of speed 60 mm/s. *n* = 13 mice for WT group; *n* = 15 for *Mecp2*-null group. *P < 0.05, ***P < 0.001, RM-ANOVA test. ns, not significant.

We placed symptomatic 7- to 8-week-old, head-fixed, male *Mecp2*-null mice and their WT littermates on a wheel whose speed is controlled by an experimenter (Fig. 1A). We analyzed forelimb kinematics while the mice adapted to four different speeds (15, 30, 45, 60 mm/s). Mice started at rest (which we will call “speed 0”), then the speed was increased by 15 mm/s every 2 min up to 60 mm/s, followed by a 2-min rest period, and then the speed was set to 60 mm/s and decreased by 15 mm/s every 2 min down to 0 mm/s (Fig. 1A, right). This experiment was performed daily over 12 to 14 consecutive days. Because the limbs of head-fixed mice do not support the full weight of the body, the range of motion of the forelimbs was quite large. [This could make the task more challenging, but by relieving the mice of the need to support their full body weight, it might also help the null mice, who can suffer exercise fatigue (*23*).] We used DeepLabCut (*24*) to train a deep neural network to track several parameters for each step cycle (Fig. 1B and fig. S1A). The movement of each paw is automatically tracked by the algorithm. Step number is reported for the left forepaw, contralateral to the site of imaging in area M1 on the right, but both front paws showed similar movements. The most reliably detected features of each step were the rising phase of a spike in the horizontal plane (*x*) and a complete spike in *y* (the vertical plane), and these were used in the analysis to represent each step (Fig. 1C).

The *Mecp2*-null mice had great difficulty adapting to the moving wheel: Their limbs repeatedly “froze” (fig. S1B, indicated by the red arrows), possibly because of either muscle weakness (*23*), the apraxia that is seen in RTT, or anxiety from being on an unpredictable wheel. Because of this, *Mecp2*-null mice took fewer strides than their WT counterparts at all speeds tested, but the magnitude of the change in stride number from lower to higher speeds was very similar between the two genotypes (an increase of approximately 70% from 15 to 60 mm/s; WT: 15 mm/s, 271.8 ± 25.1; WT: 60 mm/s, 475.7 ± 23.7; null: 15 mm/s, 146.0 ± 9.5; null: 60 mm/s, 261.5 ± 12.5; Fig. 1D). As the WT mice developed skill on the wheel, they took fewer (538.8 ± 29.6 on day 2 versus 434.1 ± 20.9 on day 10) but slightly longer strides (4.1 ± 0.2 mm on day 2 versus 5.0 ± 0.3 mm on day 10) (Fig. 1, E and F). This pattern suggests that, in WT mice, the process of learning to adapt to the running wheel takes about 1 week (the early learning phase) and that the learned skill is consolidated in the second week (consolidation phase). The null mice did not show a significant reduction in stride number (perhaps because moments of “freezing” skewed the average), but their average stride length increased steadily over the course of training (Fig. 1, E and F). The most notable change, however, was that the *Mecp2*-null mice developed much better forelimb coordination, reflected by the increased negative correlation of the traces of the two front paws (Fig. 1, G and H, and movies S1 and S2). Similar to the WT mice, the null mice showed greater changes during the first week of training than the second week (fig. S1C), suggesting that they follow a similar pattern of learning and consolidation, although they continued to improve during the second week (Fig. 1H). It may be that loss of MeCP2 extends the period required for motor skill learning but does not abolish it. We next investigated how the motor circuit might be reshaped by this training.

### M1 neurons in *Mecp2*-null mice are hypoactive but retain plasticity

**Fig. 2.**
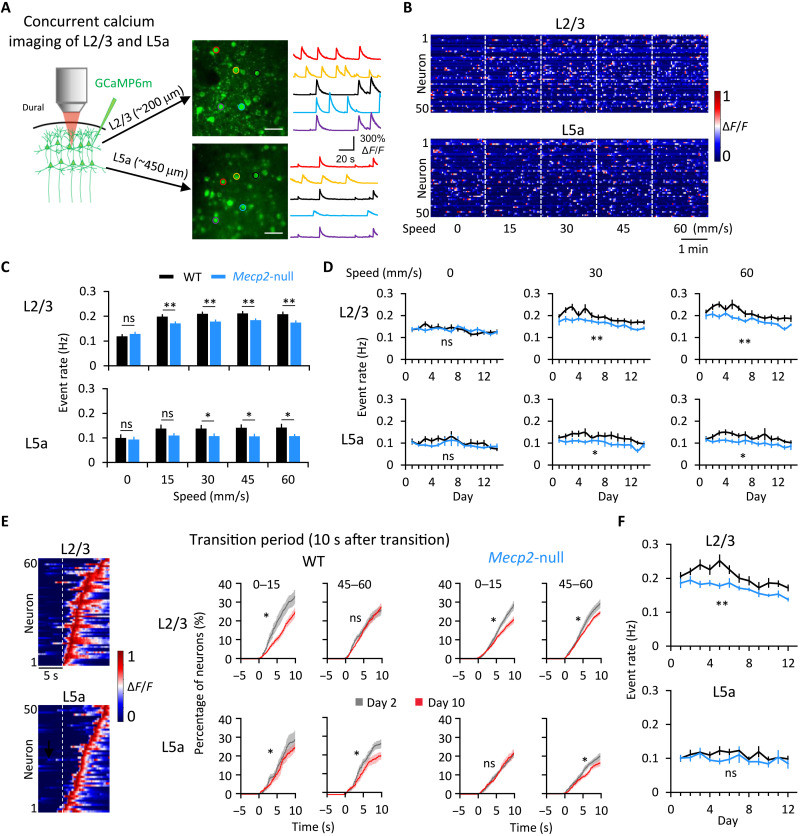
Motor learning streamlines distribution of event rates in L2/3 and L5a. (**A**) Images of pyramidal neurons expressing GCaMP6m in L2/3 (top) and L5a (bottom) of one mouse. The color of the circle around a given neuron in the photo matches the color of its activity trace. Scale bar, 50 μm. (**B**) Heatmap of neuronal activity from 50 representative neurons (rows) during increasing-speed mode. Colors represent normalization to the highest fluorescent intensity change (Δ*F*/*F*) of each neuron. (**C**) Averaged event rates of the detected neurons at five speeds. *P < 0.05 and **P < 0.01, RM-ANOVA test. (**D**) Averaged event rates across training days. L2/3 WT: *n* = 13; L2/3 *Mecp2*-null: *n* = 14; L5a WT: *n* = 11; L5a *Mecp2*-null: *n* = 13. *P < 0.05 and **P < 0.01, RM-ANOVA test. (**E**) Left: Heatmap of neuronal activity around a speed transition from 0 to 15 mm/s from one WT mouse. Right: Cumulative proportion of transition-associated neurons from all mice, on days 2 and 10, sorted by the peak of their response during 10 s after the transition from 0 to 15 and 45 to 60 mm/s. *P < 0.0001, Kolmogorov-Smirnov test. (**F**) Averaged event rate of neurons during 10 s after five speed-increasing transitions on each day of training. L2/3 WT: *n* = 11; L2/3 *Mecp2*-null: *n* = 12; L5a WT: *n* = 8; L5a *Mecp2*-null: *n* = 10. RM-ANOVA test. Error bars represent means ± SE. **P < 0.01.

Although ex vivo experiments show that cortical pyramidal neurons lacking *Mecp2* are hypoactive (*25*), we wondered how M1 pyramidal neurons behave in vivo during movement. In particular, we wanted to know how the interaction between the pyramidal neurons in L2/3 and L5a is refined by motor training. Previous studies have been limited by the inability to record neurons in the two layers simultaneously, but, here, we used a specialized two-photon microscope equipped with high-speed piezo-Z optical axis objective positioner that was able to accomplish this feat (Fig. 2A). We confirmed the proper depth for L5a with the L5-specific Rbp4-Cre line (fig. S2A, top) and did not observe obvious depth differences between the WT and *Mecp2*-null mice (fig. S2A, bottom). Thus, although the greater depth of L5a diminishes the quality of recording, it does so to the same degree in both genotypes and so did not affect comparisons between the two mouse lines. L5a somata were notably larger than those of L2/3 (fig. S2B), confirming the different types of neurons imaged in these two layers.

We identified the fluorescent transients (events) during the task (fig. S2, C and D) and analyzed the event rate of the same groups of pyramidal neurons in the right forelimb area of the primary motor cortex (M1) across the 14 days of imaging. In WT mice, the mean event rate in both L2/3 and L5a was higher during running than at rest (Fig. 2, B to D, and fig. S3). Null mice showed similar L2/3 and L5a event rates as WT at rest and 15 mm/s, but both layers were less active at all other speeds (Fig. 2, C and D). The differences did not depend on the direction of change (i.e., increasing or decreasing from the previous block; figs. S3 and S4). The event rate of L2/3 neurons fell during running by about 15 to 20% after learning in L2/3 (from 0.23 ± 0.01 on days 1 and 2 to 0.20 ± 0.01 on days 9 to 10 for WT, *P* = 0.010; and from 0.21 ± 0.01 on days 1 and 2 to 0.17 ± 0.01 on days 9 and 10 for the null group, *P* = 0.023; Wilcoxon signed-rank test), but not in L5a (from 0.11 ± 0.01 on days 1 and 2 to 0.12 ± 0.01 on days 9 and 10 for WT, *P* = 0.779; and from 0.11 ± 0.01 on days 1 and 2 to 0.10 ± 0.01 on days 9 and 10 for the null group, *P* = 0.241, Wilcoxon signed-rank test test) (fig. S5A). The overall distribution of event rates shifted toward lower rates after the learning phase, and the peak narrowed at both rest and running states (fig. S5, B and C), reflecting less variation in stimulus coding. The evolution of event rates corresponded with the mouse’s development of skill on the running wheel (Fig. 1), consistent with previous findings, demonstrating less cortical activation when executing a well-practiced, as opposed to new, motor behavior (*26*–*29*).

We next examined whether the M1 circuit in WT or null mice responded to changes in wheel speed. We found that a portion of the L2/3 and L5a ensembles responded with a higher event rate in the 10-s period after the change than in the 5-s period before the change (Fig. 2E). Comparing these transition-associated responses at each transition between day 2 (learning phase) and day 10 (consolidation phase), we found that, after learning, ~20% fewer L2/3 neurons in both WT and *Mecp2*-null mice responded to the initial transition from rest (speed 0) to 15 mm/s, and fewer L5a neurons (~25% for WT and ~15% for *Mecp2*-null mice) responded to transitions between running speeds (Fig. 2E). Overall, the difference was greatest from 0 to 15 in L2/3 for both animals, but the evolution of learning-induced changes in the response to speed changes was similar between WT and *Mecp2*-null mice (Fig. 2E). The event rates of *Mecp2*-null L2/3 neurons, but not L5a, were significantly lower than WT during the10-s period after a speed change (Fig. 2F). When we identified specific neurons and followed them over multiple days (fig. S6A; see Materials and Methods) to examine how they respond to speed changes, we found that they were exceptionally plastic: A given neuron might respond (or not) to a transition on any given day (fig. S6, B and C). This plasticity of response to a transition was at the same level in the WT and *Mecp2*-null mice (fig. S6D). The response to speed changes thus takes place at the level of the ensemble, not the individual neuron.

### Motor learning strengthens functional connectivity for a small proportion of neuronal pairs

Information is encoded at the circuit level through the coordinated firing patterns of neurons in the ensemble (*30*). We therefore analyzed the functional connectivity between recorded neurons in the M1 network throughout training using correlation analysis (*31*). Functional connectivity is commonly estimated by Pearson correlation coefficients, which include direct and indirect correlations. Direct correlation reflects functional connectivity within the local circuit by excluding shared input from afferent brain regions (*8*).

**Fig. 3.**
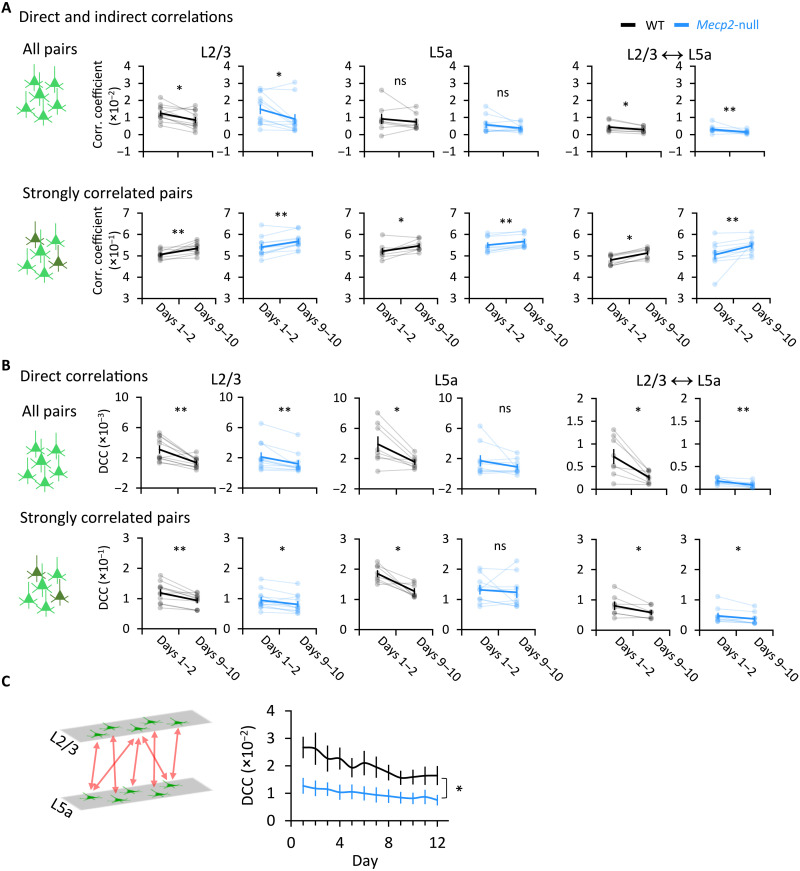
Overall functional connectivity across M1 decreases with learning, but a small percentage of neuronal pairs become more strongly correlated. (**A** and **B**) Mean direct and indirect correlation coefficients calculated as Pearson correlation coefficients (A) and direct correlation coefficient (DCC) calculated using direct correlation analysis (DCA) (B) between pairs of neurons within L2/3, within L5a, and across both layers during four running blocks for an individual WT or *Mecp2*-null mouse (top: all pairs of correlation; bottom: strong correlations, defined by a correlation coefficient that was greater than twice the SD). Light lines connect data from each individual mouse; dark lines connect the averaged data from all mice. L2/3 WT, *n* = 11 mice; L2/3 *Mecp2*-null, *n* = 12 mice; L5a WT, *n* = 8 mice; L5a *Mecp2*-null, *n* = 10 mice (we excluded the L5a data from mice from which the number of neurons detected in L5a was less than 20). Across both layers, WT, *n* = 8 mice; *Mecp2*-null, *n* = 10 mice. Error bars represent means ± SE. *P < 0.05, **P < 0.01; Wilcoxon signed-rank test. (**C**) DCC of neurons across M1 L2/3 and L5a. WT, *n* = 8 mice; *Mecp2*-null, *n* = 10 mice. Error bars represent means ± SE. *P < 0.05, RM-ANOVA test.

We first calculated the Pearson correlation coefficient between each pair of neurons within L2/3 and L5a, as well as across these two layers in both WT and *Mecp2*-null mice. At rest, the correlation coefficients of neuronal pairs did not differ between the learning and consolidation phase, with the exception of L2/3 neurons in the null mice (fig. S7A). There were no differences in correlations either within or between layers at any running wheel speeds from learning (days 1 to 2) to consolidation (days 9 to 10) (fig. S7B). However, in both genotypes, the L2/3 neuronal pairs were less functionally connected during the consolidation phase. The overall functional connectivity of neuronal pairs during running, both within L2/3 and across the two layers, diminished with training (Fig. 3A, top row). The distribution of correlation coefficients was rather broad (between −0.5 and 1) across the population of M1 neurons, and shifts in the distribution of pairwise correlations can be seen across training (fig. S8). However, in the 5% of neuronal pairs that were most strongly connected—i.e., those in which the Pearson correlation coefficient was larger than the mean plus two times the SD of the correlation coefficient—functional connectivity actually increased with training (Fig. 3A, bottom row, and fig. S8). These results suggest that refining a motor skill (in this case, adapting one’s gait to different speeds of a surface controlled by someone else) increases functional connectivity between already-correlated neurons, with an overall net decrease in functional connectivity across the rest of the population, within L2/3 and across L2/3 and L5a. This circuit refinement occurred in the *Mecp2*-null mice as well.

To verify that the changes we detected in the M1 circuit bore a causal relationship with motor training, we also imaged age-matched training-naïve mice during 5 min of free running (i.e., on a frictionless wheel) on two separate days a week apart. There was no change in the correlation coefficients of any neuronal pairs in mice of either genotype between days 1 and 8 (fig. S9A). We then imaged trained mice of both genotypes during a free-run mode before training on days 1 to 2 and 9 to 10. The correlation coefficients dropped significantly for L2/3 neurons in both WT and *Mecp2*-null mice and across both layers in *Mecp2*-null mice (fig. S9B). These data demonstrate that the changes observed in M1 result from motor learning, not merely from the activity of running or from the passage of time.

To determine whether the heightened functional connectivity of certain neuronal pairs might be due to shared input from afferent brain regions, we used a maximum entropy-based inference method [direct correlation analysis (DCA)] that has been used to infer direct interactions from biological datasets such as gene expression data or sequence ensembles while excluding indirect interactions (*32*–*34*). We calculated the averaged direct correlation coefficient (DCC) and found that the DCC decreased with training for all pairs, including strongly correlated pairs, within L2/3, within L5a, and across these two layers (Fig. 3B, top row). The magnitude of the decrease was slightly smaller in the *Mecp2*-null mice than in WT mice. This indicates that the functional connectivity among L2/3 neurons decreased over the course of learning. The data also indicate that the greater temporal correlation of firing among a small proportion of L2/3 neurons (Fig. 3B, bottom row) was due to more synchronized input to these neurons from other brain regions (most likely the somatosensory and premotor cortex) over the course of learning (*8*). Note that, after learning, there was no difference between genotypes in direct correlation (fig. S10), but cross-layer connectivity between L2/3 and L5a was much lower in *Mecp2*-null mice than in WT mice (Fig. 3C), reflecting a profound disruption of interlaminar functional connectivity in these animals.

In summary, functional correlations between strongly correlated neuronal pairs increase with learning, likely due to synchronized input from the somatosensory and premotor cortices. This small portion of strengthened functional correlations takes place against a background of overall diminishing functional correlations within the ensemble. This suggests that the ensemble becomes more efficient by recruiting the same small group of neurons to the task.

### MeCP2-deficient neuronal pairs are less stable than WT and do not show proximity bias

**Fig. 4.**
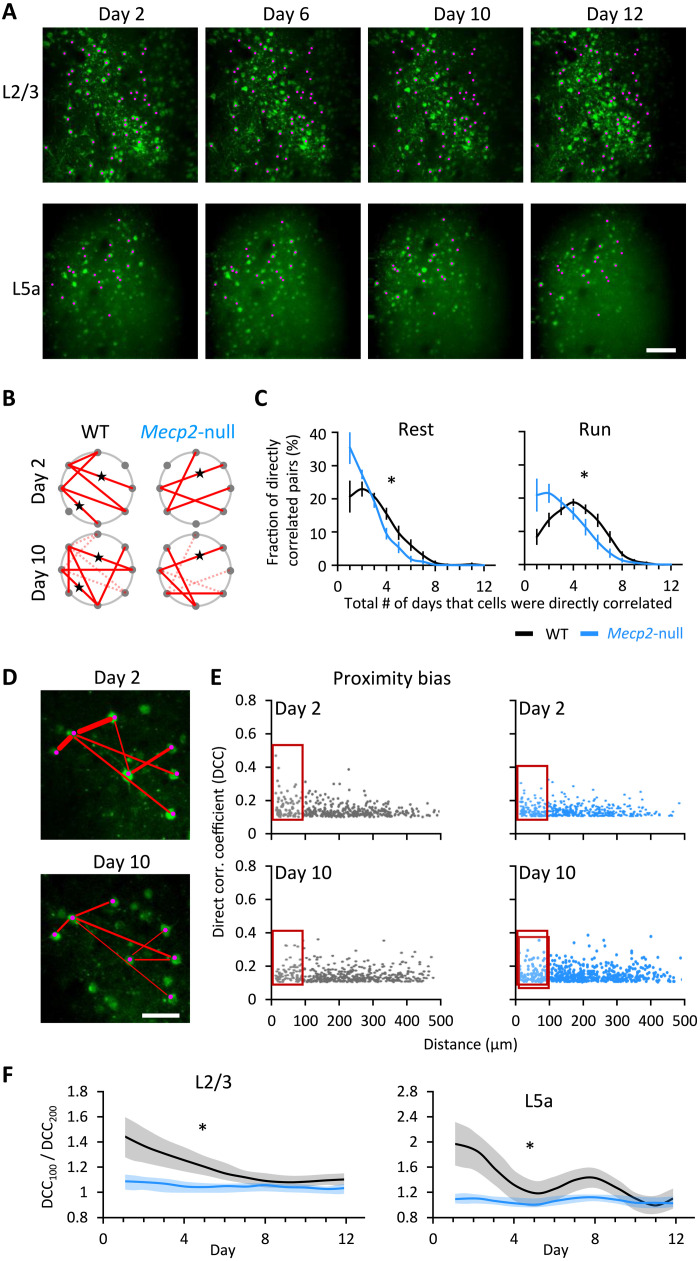
Loss of *Mecp2* shortens the duration of synaptic connections during learning. (**A**) Images of the same field of view on L2/3 and L5a on different days from a WT mouse. Pink dots indicate the same active neurons detected on multiple days. Scale bar, 100 μm. (**B**) Diagram demonstrating direct correlation between neurons on day 2 (top) and day 10 (bottom) of WT (left) and null mouse (right). Each dot represents one neuron. Red lines indicate direct correlations between two neurons. Dashed lines indicate connections that disappeared from days 2 to 10. Stars label the connections that existed on both days (there were fewer such connections in null mice than in WT mice). (**C**) The percentage of the directly correlated neuronal pairs that were detected over a different number of days across the 2-week testing period at rest (left) and running (right) state. WT, *n* = 9 mice; *Mecp2*-null, *n* = 9 mice. Error bars represent means ± SE. *P < 0.05, *t* test between the kurtosis values that represent the shape of two curves. (**D**) Direct correlations between neurons in the same field of view on days 2 and 10. Red lines represent direct connectivity between a pair of neurons. Line thickness represents correlation strength. Scale bar, 50 μm. (**E**) Relationship plot between the DCC of a pair of neurons on days 2 and 10 and their physical distance from each other. *x* and *y* axes represent the distance of the paired neurons and their DCC, respectively. The red box marks the neuronal pairs, whose distance is less than 100 μm. (**F**) Ratio of DCC between neuronal pairs with a distance of less than 100 μm (DCC_100_) and between 100 and 200 μm (DCC_200_). L2/3 WT, *n* = 11 mice; L2/3 *Mecp2*-null, *n* = 12 mice; L5a WT, *n* = 8 mice; L5a *Mecp2*-null, *n* = 10 mice. *P < 0.05, RM-ANOVA test.

To further explore the effect of MeCP2 deficiency on M1 circuit plasticity, we used DCA to search for functional connectivity among the same group of neurons within each mouse. We were able to track ~20% of all active neurons throughout the 12 days using the method in fig. S6A and counted how many times we detected functional connectivity between the same pair of neurons (Fig. 4A). Strongly correlated pairs of neurons were more likely to be detected repeatedly over the 2-week period in WT mice than in *Mecp2*-null mice (Fig. 4, B and C); the persistence of a given pairing was more probable with running than rest in WT mice (Fig. 4C). Notably, most pairs were detected in WT mice over 4 days (during running), but in *Mecp2*-null mice, most neuronal pairings occurred only once or twice (during running or resting). Functional connections in both genotypes were virtually undetectable beyond 8 days. The number of pairs that formed in WT mice on only 1 or 2 days during runs was half the number found in the null; conversely, null mice had about half the number of pairs as WT mice (during rest or running) that were detected more than 4 days (Fig. 4C, right). In other words, the null mice are able to form functional connections but at half the rate and for not nearly as long. Although we were not examining actual synaptic connections, these data are in agreement with previous studies showing that physical synaptic connections are attenuated in *Mecp2*-null mice (*35*, *36*).

We next asked whether functional connectivity was influenced by physical proximity, as previous studies have observed a “proximity bias” in normal circuits (*37*, *38*). In the initial stages of motor training, we found that the closer the two L2/3 neurons were to each other in the WT brain, the higher their DCC (Fig. 4, D and E). This proximity bias was much less apparent in the null mice, similar to what we previously observed in the null hippocampal circuit (Fig. 4E) (*19*). To visualize how proximity bias evolved with training, we plotted the ratio of the DCC for pairs closer than 100 μm (DCC_100_) to pairs at a distance between 100 and 200 μm (DCC_200_) (Fig. 4F). In WT mice, this ratio declined over the first week of training and then stabilized in L2/3 and L5 (Fig. 4F, black lines). In *Mecp2*-null mice, however, the ratio started low and did not change with training (blue lines). Thus, WT mice have a proximity bias that diminishes with learning, as the strongly correlated pairs whose functional connectivity grows are not necessarily near one another. *Mecp2-*null animals have no proximity bias to begin with despite the fact that they have smaller, more closely packed neurons (*13*, *14*).

### Trained *Mecp2*-null mice are better coordinated, less anxious, and live longer

**Fig. 5.**
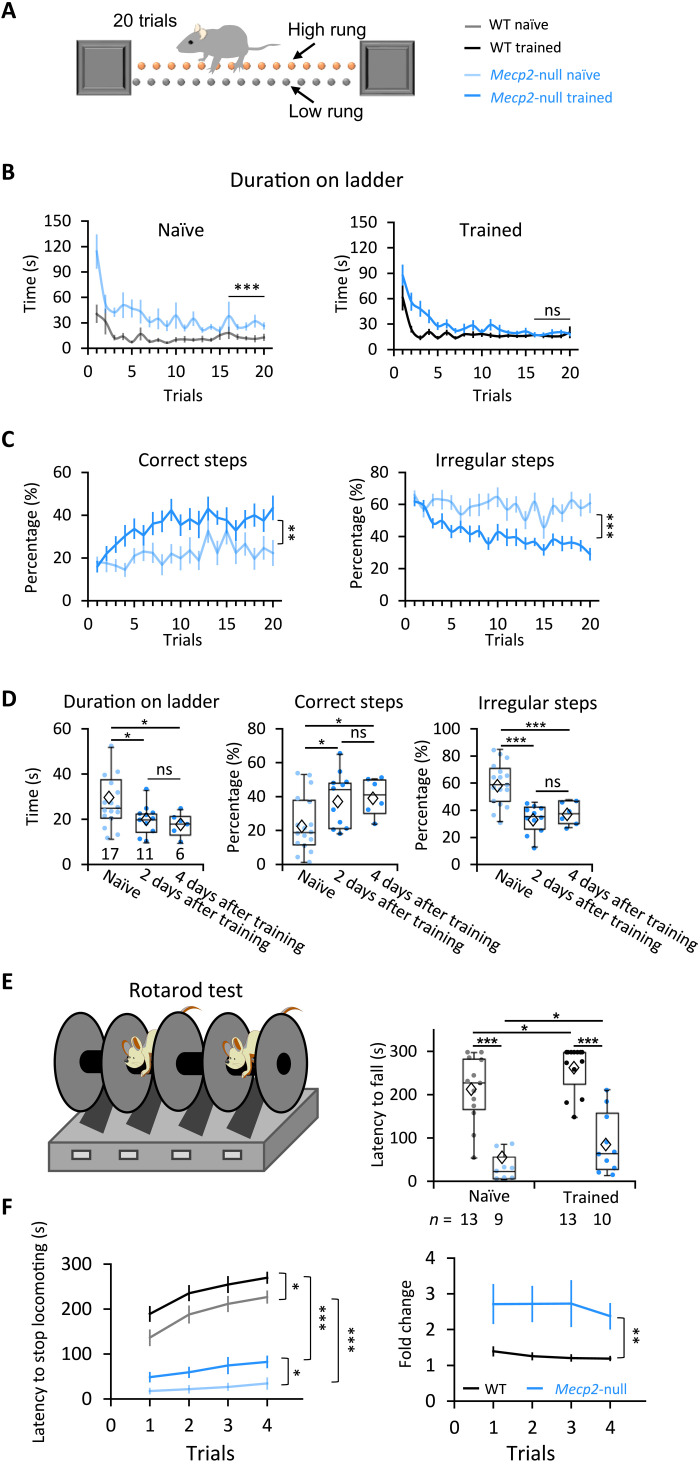
Motor learning improved coordination of *Mecp2*-null mice in different assays. (**A**) The ladder test as a measure of coordination. The animals run from one end to the other 20 times (20 trials). (**B**) How much time it took for mice to walk from one end of the ladder to the other. Naïve: WT, *n* = 15; *Mecp2*-null, *n* = 17; trained: WT, *n* = 19; *Mecp2*-null, *n* = 17. (**C**) Averaged percentage of correct and irregular steps at each trial. In (B) and (C): **P < 0.01 and ***P < 0.001, RM-ANOVA test. (**D**) Performance parameters from the last five trials of the ladder test 2 or 4 days after wheel training ended (for trained mice) or from age-matched naïve mice. Naïve: *n* = 17 mice; 2 days after training: *n* = 11 mice; 4 days after training: *n* = 6 mice. Error bars represent means (diamond) ± SE. *P < 0.05 and ***P < 0.001, one-way ANOVA test with Fisher’s least significant difference post hoc test. (**E**) Latency to fall in the first trial of the accelerating rotarod test 2 days after training. The number of mice examined is presented in each column. Error bars represent means ± SE. *P < 0.05 and ***P < 0.001, two-way ANOVA test with Sidak’s post hoc test. (**F**) Latency to stop locomotion (left) and fold change (right) (calculated as the mouse’s latency to stop divided by the mean value of the naïve mice) on the rotarod for four trials. Naïve: WT, *n* = 11 mice; *Mecp2*-null, *n* = 7 mice; trained: WT, *n* = 9 mice; *Mecp2*-null, *n* = 8 mice. Error bars represent means ± SE. *P < 0.05, **P < 0.01, and ***P < 0.001; RM-ANOVA test.

Daily running on a speed-controlled wheel is analogous to forced exercise, which has proven broadly beneficial in patients with Parkinson’s disease (*39*, *40*). Exercise has also been shown to reduce stereotypic behaviors in children with autism spectrum disorders (*41*). We therefore asked whether the variable-speed wheel training improved motor function more generally in *Mecp2*-null mice. We first examined the performance of the mice crossing a ladder over 20 trials after the last day of training on the wheel (Fig. 5A). As expected, age-matched naïve *Mecp2*-null mice had difficulty throughout the 20 trials, although they did improve. The trained *Mecp2*-null mice, however, performed similar to WT mice after about 12 trials of ladder running (Fig. 5B and fig. S11). Compared to the naïve *Mecp2*-null mice (movie S3), trained *Mecp2*-null mice made significantly more correct steps (steps between adjacent high rungs) and fewer irregular steps (i.e., those not between high rungs; see Materials and Methods) (Fig. 5C and movie S4). Trained *Mecp2*-null mice maintained their superior coordination 2 and 4 days after the last day of wheel running, indicating that benefits from assisted motor training can last at least a few days (Fig. 5D).

To see whether this improved coordination translated to other circumstances, we also tested mice on the accelerating rotarod test. (Although this test is similar to the computerized running wheel, the mice bear their own full weight.) Motor training more than doubled the latency to fall in *Mecp2*-null mice, although they still performed much more poorly than their trained WT littermates (Fig. 5E). We noticed that, instead of falling off the rod, most mice simply stopped running and held onto the rotating rod when they got tired, perhaps reflecting the fatigue to which *Mecp2*-deficient muscles are vulnerable (*23*). We therefore decided to compare the time when the animals first stopped locomotion, either by holding onto or falling off the rod. Although motor training significantly increased the latency to stop locomotion by either means, for trained mice of either genotype compared to naïve controls (Fig. 5F, left), the fold change was much more notable in *Mecp2*-null mice than that in WT mice (Fig. 5F, right).

**Fig. 6.**
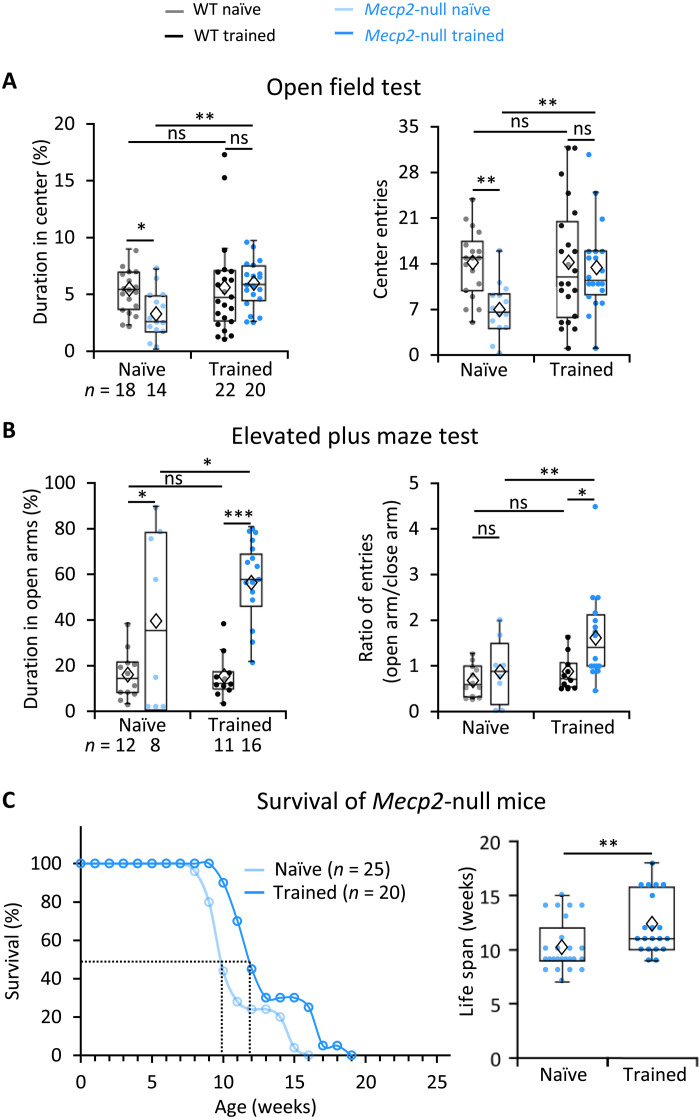
Motor training diminished anxiety and extended survival of *Mecp2*-null mice. (**A**) Performance in the open field test. Naïve: WT, *n* = 18 mice; *Mecp2*-null, *n* = 14 mice; trained: WT, *n* = 22 mice; *Mecp2*-null, *n* = 20 mice. Error bars represent means ± SE. *P < 0.05, **P < 0.01; two-way ANOVA test with Sidak’s post hoc test. (**B**) Performance in the elevated plus maze test. Naïve: WT, *n* = 12 mice; *Mecp2*-null, *n* = 8 mice; trained: WT, *n* = 11 mice; *Mecp2*-null, *n* = 16 mice. Error bars represent means ± SE. *P < 0.05, **P < 0.01, and ***P < 0.001; two-way ANOVA with Sidak’s post hoc test. (**C**) Left: Exercise training extended the survival of *Mecp2*-null mice. Right: Average life span of naïve and trained *Mecp2*-null mice in the plot at left. Naïve: *n* = 25 mice; trained: *n* = 20 mice. **P < 0.01, Mann-Whitney *U* test. In the box plots, diamonds represent the group mean.

The trained mutant mice did not show greater strength in the grip test, so their improved motor function was not due to an increase in muscle strength (fig. S12A); nor did they move faster or move at longer distances in the open field test (fig. S12B), but they spent significantly more time in the anxiogenic center area than untrained *Mecp2*-null mice (Fig. 6A). This suggests that forced motor training mitigates the *Mecp2*-null mouse’s well-documented anxiety (*15*, *42*). In another classic assay for anxiety, the elevated plus maze test, the naïve null mice spent more time in the open arms than WT mice, although they did not enter them as frequently (Fig. 6B, left) as others have observed (*42*). Trained *Mecp2*-null mice entered the open arms more frequently than the closed arms, signifying less anxiety (Fig. 6B, right).

Last, the trained mutant mice survived ~20% longer (from 10 to 12 weeks; naïve: 10.2 ± 0.5 weeks versus trained: 12.3 ± 0.6 weeks; *P* = 0.002, Mann-Whitney *U* test; Fig. 6C). We conclude that forced motor training can improve coordination, rescue anxiety, and extend the survival of *Mecp2*-null mice. When we analyzed the relationship between survival time and improved performance on different assays, we found no correlation between life span and better performance on the rotarod or anxiety tests, but we did find a weak correlation (*P* = 0.236 and *r* = 0.336; fig. S13) between coordination and life span. This suggests that motor learning may contribute to extending the life span of *Mecp2*-null mice beyond the benefits of exercise itself. This would align with previous studies indicating that environmental enrichment benefits RTT mice (*43*–*45*).

## DISCUSSION

The idea that practice enables the refinement of motor skills is uncontroversial, but the motor circuit changes that underlie this intuitive principle have been challenging to elucidate. Our results support a model in which motor skills are learned by judiciously strengthening the functional connectivity of a subgroup of neurons to store information while decreasing connectivity among the rest of the population to maintain flexibility for learning new skills (*46*). This reorganization is attenuated but not abolished by loss of MeCP2. The motor circuit in RTT, although hypoactive and less able to maintain synaptic connections, retains sufficient plasticity to support learning, although the entire brain is disturbed by loss of MeCP2 in these animals.

It has been proposed that individual neurons increase their ability to discriminate similar stimuli through three processes during motor learning: sharpening their tuning curves, gain modulations, and peak shift (*47*, *48*). Previous studies on primate motor and sensory cortices revealed that motor learning is restricted to a subgroup of neurons (*49*, *50*). Early training modulates the activity of both the target neurons and nearby cells; as training progresses, only the activity of the target neurons is modulated. Our observation that motor training strengthened the functional connectivity of a small proportion of neurons is consistent with these studies and supports the recently proposed notion that motor learning leads to refinement of cortical population dynamics so that more reliable neural trajectories produce skilled behavior (*51*). However, only the indirect, not direct, correlation coefficients of strongly correlated pairs increased. We hypothesize that this change in functional connectivity is due to a specific increase in the strength of the input from the somatosensory cortex (S1) to L2/3. The main inputs to L2/3 of M1 are from the somatosensory cortex and premotor cortex (*52*), but motor learning in the present study depends to a much greater degree on somatosensory feedback coming from S1 rather than volitional inputs from the premotor areas. Moreover, the L2/3 M1 neurons do not respond specifically to running speeds or speed transitions, suggesting that their activity may be more related to somatosensory feedback and other task-related signals. Further investigation is needed to determine whether afferent axons from S1 form more connections with the highly connected M1 neurons after learning.

A recent study that recorded L2/3 neurons in mice grasping for food on a rotating wheel concluded that these neurons respond to success or failure in the task, rather than specific elements of the movements themselves, to reinforce skill learning (*53*). It may be that the neurons we recorded as having a higher firing rate during the 10 s after speed transitions were responding to the unexpected change in the environment as a “failure” to match the speed of the wheel. We did find that a certain proportion of neurons were active even a minute past the transitions, but our experimental setup, which was continuous running, without reward or pauses when the task was completed successfully, did not allow us to analyze whether the circuit recorded speed transitions as mistakes of some sort.

One of the most notable clinical features of RTT is the loss of acquired skills in the second or third year of life. Acquired motor and language skills can disappear in a matter of days to weeks. At a behavioral level, the children seem unable to retain what they have previously learned or to learn new skills. Whatever roles MeCP2 has in early development (*54*), it seems to have additional functions in neuronal or synaptic maintenance (*11*). The period between 10 and 15 weeks of age is one of heightened plasticity, when we see elaboration of dendritic arbors and elevated expression of synaptic proteins; these processes seem to be profoundly disrupted in the absence of MeCP2 (*55*, *56*). Despite these defects, the null mouse motor circuits showed plasticity in L2/3 (Fig. 2) and across layers (Fig. 3) in a similar pattern as in WT animals. The new synaptic connections initiated by null L2/3 and L5a neurons, however, tended not to last as long, which further supports the notion that MeCP2 is involved in neuronal maintenance. The number of these short-lived functional connections in null mice was over twice the number observed in WT animals (Fig. 4B), although this could be because the greater density of neurons on the null mice allowed us to record many more neurons. These observations call to mind an earlier study of the visual circuit in *Mecp2*-null mice. Noutel *et al*. (*57*) found that the initial development of synapses between retinal ganglion cells and relay neurons in the dorsal lateral geniculate nucleus proceeded normally, but that synapses failed to be strengthened during the subsequent period of visual experience-dependent plasticity of the *Mecp2*-null mice. A previous ex vivo study using glutamate uncaging and laser scanning photostimulation in M1 slices showed that knocking down MeCP2 reduces connectivity between M1 L2/3 and L3/5a neurons (*35*). Dani and Nelson (*36*) used paired recordings to reveal that there were fewer connections to L5 pyramidal neurons from the somatosensory cortex of *Mecp2*-null mice; moreover, individual connections were weaker. These results using slice recording align well with our findings that the connectivity of M1 pyramidal neurons is reduced in *Mecp2*-null mice.

The current study shows that 2 weeks of training reduced anxiety and extended the life span of symptomatic *Mecp2*-null mice by 20%. We had previously shown that DBS rescues hippocampal-dependent learning and memory in RTT mice (*18*) because it corrects hippocampal circuit abnormalities (*19*). DBS provides a very strong stimulus, at around 130 Hz, which is considerably higher than the normal firing rate for neurons. Wheel running is a much more “physiological” stimulus and still is sufficient to alter circuit function and behavior of symptomatic *Mecp2*-null mice. While this manuscript was in revision, Achilly *et al*. (*58*) published a study showing that exercise benefits female *Mecp2*-heterozygous mice that are not yet symptomatic. They also found some benefit for mice that were trained after becoming symptomatic, although they de-emphasize this result. However, their mice did not appear to benefit in other realms, so they conclude that benefits of exercise are task specific. We suspect that the broader benefits we observed came from our different training strategy. First, we used a forced exercise paradigm; otherwise, symptomatic *Mecp2*-null male or heterozygous female mice are too sick to train. The fact that the head fixation prevented the mice from having to support their full body weight probably allowed them to train more intensively than they could on the accelerating rotarod. [Even our trained mice stopped running on the rotarod when they got tired; we suspect exercise fatigue (*23*) or apraxia would have been a limiting factor in the study of Achilly *et al*. (*58*), which used the rotarod for training]. Second, our circuit-level analyses suggest that mutant mice would learn even better with daily training than with the biweekly rotarod training regimen used in (*58*).

We also suspect that the additional benefits we observed (diminished anxiety and increased life span) involve motor learning as distinct from exercise per se. Exercise alters multiple neural circuits through stimulating brain-derived neurotrophic factor and many other changes, as has been documented in many studies (*40*, *44*, *45*, *59*), and the fact that rotarod training in (*58*) delayed the onset of disease symptoms in RTT mice speaks powerfully in favor of exercise. In favor of our hypothesis, however, we found a positive although weak correlation between extension of life span and improvement in forepaw coordination. Note in this context that Achilly *et al*. (*58*) found that training in the Morris Water maze, which arguably involves more learning than exercise, led to improvements in the morphology and function of hippocampal neurons. Their Morris water maze training regimen (4 days/week) may have improved cognition because it works within the 2-day window of functional connections we observed, if we assume that other *Mecp2*-deficient pyramidal cells behave in the same way as those in M1, which may not be the case. Regardless, the improved dendritic arborization they observed in hippocampal neurons from water maze–trained mice is consistent with a hypothesis that learning may contribute benefits that are distinct from those of exercise.

There have been a few case reports and small studies showing girls with RTT benefit from exercise (*60*–*64*), and the current study certainly confirms that these reports should not be dismissed as anecdotal. Early environmental enrichment in RTT mouse models has also reduced various behavior deficits (*44*, *45*, *65*, *66*). This suggests that stimulation in general—in other words, exercise and learning of all sorts—is beneficial in individuals with RTT, as it is in healthy humans.

## MATERIALS AND METHODS

### Animals

Mice were housed in an American Association for Laboratory Animal Science–certified level 3 facility on a 14-hour light cycle. Male *Camk2-cre; Mecp2^−/y^* (null) mice and female *Camk2-cre; Mecp2^−/+^* mice were obtained by breeding heterozygous female *Mecp2^+/−^* mice on the 129S6/SvEvTac strain (*20*) (a gift of H. Zoghbi) to male *Camk2-cre* mice carrying the *Camk2-cre* allele on the C57/B6 strain obtained from the Jackson Laboratory (JAX, #005359). All procedures to maintain and use these mice were approved by the Institutional Animal Care and Use Committee for the George Washington University.

### Surgeries

At the age of 4 weeks, male mice were deeply anesthetized and then prepared for stereotaxic surgery. A 3-mm-wide hole was drilled through the skull over the motor cortex. The center of the craniotomy was ~1.6 mm lateral to bregma (*67*). AAV/DJ-flex-GCaMP6m virus was injected into the forelimb area of the right motor cortex [1.5 mm lateral and 1.5 mm anterior to bregma, according to previous studies (*4*)] at two different depths (200 and 450 μm) to reach L2/3 and L5a neurons, respectively, via a 1.0-mm optical density glass microneedle with a 10- to 20-μm-diameter tip attached to a Nanoject microinjector pump. A glass coverslip was then placed over the exposed brain and sealed into the skull. The surgical site was closed using a veterinary surgical glue. A 2-g titanium headpost washer was attached to the head with “cold cure” denture material for later head fixation under a two-photon microscope.

To verify imaging depth and location of viral expression, three animals were perfused with 10% formaldehyde, and the brains were sectioned. The depth for L5a was confirmed with three Rbp4-Cre mice as shown in fig. S2A.

### Chronic in vivo two-photon imaging

The experimental animal was lightly sedated with isoflurane and placed head-fixed to the microscope, on a wheel, and then allowed to wake up. The mouse was acclimated to the wheel in the free-running mode for 5 min, head-fixed under a Thorlabs Bergamo II two-photon microscope (Thorlabs, NJ) with high-speed piezo-Z optical axis objective positioner and a Nikon 25× (numerical aperture of 1.1) water-immersion objective lens (Nikon Instruments Inc., NY). Time-lapse images were acquired with Thorlabs software at 6 to 7 frames/s per layer for 4630 frames per layer for 12 min (2 min for each speed for both speeding up and slowing down modes). The interval between the two modes at each trial was 2 min. The laser power was adjusted to the minimum, necessary to achieve ideal fluorescence intensities during each imaging session. On average, about 20 to 30 mW of power arrives at the sample at L2/3 and 120 to 160 mW of power at L5a. In WT mice (*n* = 13), we imaged a total of 28,125 L2/3 neurons and 10,395 L5a neurons; in *Mecp2*-null mice (*n* = 14), we imaged a total of 44,585 L2/3 neurons and 20,545 L5a neurons. The reason for the greater number of recorded neurons in the null mice is that they have smaller, more densely packed cells. The ratio of null to WT neurons recorded in this study is ~1.47, which is very close to the 1.5 derived from an earlier study (*14*) that found that the cell density of L2/3 neurons in the somatosensory cortex of *Mecp2*-null mice after 8 weeks of age is about 120 neurons/10^5^ μm^2^, while it is only about 80 neurons/10^5^ μm^2^ in WT mice.

### Setup for studying motor skill learning over a 2-week period

Beginning at 4 weeks after surgery, experimental animals were trained daily for 14 consecutive days on a computerized wheel system customized by Delta Commercial Vision (NJ, USA), while two-photon imaging was performed simultaneously (Fig. 2A). The 6-inch-diameter foam wheel is connected to a motor controlled by an Adreno chip connected to a computer via a USB cable so that the experimenter can control the speed of the wheel rotation. There are five speeds—0, 15, 30, 45, and 60 mm/s—and the experiment maintained each speed for 2 min, either in ascending or descending order.

Before the start of each trial, the experimental animal was put on the treadmill with the head fixed at a comfortable height under the two-photon microscope. ThorCam software controlled the videotaping of the mouse at 71.5 Hz via a high-speed camera (Thorlabs, NJ) and simultaneously triggered the ThorImage software (Thorlabs, NJ) to start two-photon imaging and the digital command that controls the treadmill. The experimental task is to learn to smoothly and quickly adapt to running at different speeds.

Each trial comprised three sessions. “Session 1” was 12 min in total. It started at speed 0 (rest) and increased by 15 mm/s every 2 min up to 60 mm/s, followed by a 2-min rest period. “Session 2” was a 2-min interval for the animal to rest and for the experimenter to save the data. “Session 3,” also 12 min in total, started at speed 0, jumped to 60 mm/s and remained there for 2 min, and then decreased by 15 mm/s every 2 min down to 0 mm/s. There was one trial per day for 2 weeks.

### Motion analysis

We detected paw positions with an efficient pose estimation method called DeepLabCut, which is based on transfer learning with deep neural networks. We chose 3 days from the 14 days of experimental data and chose the period at speed 60 for training. We next used five body markers to track forelimb movement. We tracked five points: the center of the two forepaws, two front knees, and the center of the chest to form a “skeleton.” For every training image, we dragged each skeleton point to the appropriate body part, and the program saved the label positions automatically. With limited training data, we achieved a satisfying model to run on all movies and detect paw locations. Then, we saved the coordinates of all the body markers and used the Butterworth filter to process the raw data with order 2 to reduce the influence of error.

To get the paw location value (*d*), we first chose the median of the paw coordinates as the zero point. We gave forward paw positions positive position values and backward paw positions negative position values

$$d_{\text{new}}=\alpha (d_{\text{median}}-d_{\text{raw}})$$

where *d*_raw_ is the original distance between the forepaw’s location and the lower right corner, *d*_median_ is the median of the distance, and α is a parameter used to convert pixels into millimeter.

According to the location of the paw, the start and end point will be detected by an algorithm. As shown in Fig. 1C, the *x* and *y* axis represent the direction of paw movements along the horizontal and vertical planes, respectively. Stride was represented by the rising phase of a spike in *x*; a complete spike in the paw coordination index was defined as the negative value of the Pearson correlation coefficient of the two paw location traces.

### Calcium image processing

Calcium images were processed and analyzed using custom scripts in Python based on CaImAn (*68*, *69*), a toolbox for large-scale calcium imaging data analysis and behavioral analysis. This toolbox provides fast and scalable algorithms for motion correction, source extraction, spike deconvolution, and component registration across multiple days.

Source extraction was performed using the online algorithms (*69*) by adapting constrained nonnegative matrix factorization approaches (*68*, *70*). For the prediction of spikes from fluorescence traces, we applied unsupervised learning sparse nonnegative deconvolution (*71*) and near-online Online Algorithm for Scalable Image Similarity (OASIS) algorithm (*72*) to our raw calcium signals. To align calcium recordings collected from speed-increasing and speed-decreasing modes, we first artificially stacked all calcium videos collected in one trial for the same mouse, and then CaImAn performed motion correction using the NoRMC algorithm (*73*), which corrects nonrigid motion artifacts by estimating motion vectors with subpixel resolution over a set of overlapping patches within the field of view (FOV). The width of event rate distribution is calculated as full width at half maximum of the distribution by the function scipy.signal.peak_widths in the scipy package in Python.

### Detecting the same neuron across trials

Although we imaged the same area of the motor cortex for each experiment, little shifts do occur in the FOV between trials. Thus, both the total number and precise location of detected neurons can vary somewhat across trials. To accommodate this slight inconsistency and enable direct comparison between trials for each mouse, we aligned the images between days and picked the same neurons throughout 14 trials. The basic workflow is shown in fig. S6A.

First, we constructed an image of the distribution of neurons based on their pixel location. Instead of representing a neuron in a single pixel, we used a bivariate normal distribution peak centered at its location with a maximum height of 1.0 and SD as 5, which is around the radius of real neurons in the frame. The reconstructed image has the same size as the raw frame (512 by 512), as shown in fig. S6A. For each sample (from each trial), we used the detected neuron from trial 1 as a reference and aligned all 14 images (including subsequent trials). To do this, we expanded the image of trial 1 slightly so that neurons that would otherwise appear slightly “displaced” from the first image became aligned with the selected neuron; we created a border around the image with a width of approximately 80 pixels to accommodate the maximum shift we observed between day 1 and any other trial (and prevent the program from having nothing to compare at the edges of the FOV). For each map, we calculated the correlation score of the two-image matrix (day 1 × day *n*), resulting in a correlation matrix of 81 × 81 (fig. S6A, step 5). Correlation matrices have a size of $\left(\frac{2W}{S}+1)\times (\frac{2W}{S}+1\right)$; the shift (Δ*x*, Δ*y*) with the highest correlation score indicates the degree of shift of the image from day 1 to the present image. A desired correlation matrix should be sparse, i.e., except for a very small region with high correlation score, most parts should be nearly zero.

Given a known degree of shift between two trials, it becomes straightforward to map the same neuron by correcting the location of neurons from the subsequent trial and matching to day 1. Considering the various shapes and sizes of neurons, we allowed for a difference of 5 pixels when matching. Thus, for each neuron *N*_i_(*x*_i_,*y*_i_) at day 1, we searched for the same neuron on the subsequent trial image, and if there was a neuron within 5 pixels of (*x*_i_,*y*_i_), then we assumed that it was the same neuron; otherwise, we recorded no corresponding neuron on that trial’s image. If there was more than one neuron within 5 pixels, then we chose the one closest to the original *N*_i_. Figure S6A (step 6) shows examples of matched neurons over two different days. On average, 20% of the total active neurons detected were consistently recorded each day throughout 12 days.

### Identification of neurons responsive to speed transitions

We identified neurons particularly responsive to speed transitions based on their response each time the computerized wheel transitioned from one speed to another, in either session, following the steps below:

1) For any neuron *k*, we calculated the similarity between the original calcium trace (Δ*F*/*F*) *c_k_* and the speed transition vector *s*, where *s_t_* = 1 if time *t* was within 15 s of every speed transition, otherwise *s_t_* = 0. The similarity was defined as the normalized inner product of two vectors, 2*s* · *c_k_*/(∣*s*∣^2^ + ∣*c_k_*∣^2^). The value of the similarity was between 0 and 1. A value of 1 meant that these two vectors were identically distributed, while a value of 0 meant that these two vectors were completely different.

2) We then randomly shuffled the speed transition vector *s* 5000 times and calculated the similarity between *c_k_* and *s*_shuffled_ to generate a similarity distribution representing the similarity histogram predicted by chance for neuron *k*.

3) Neuron *k* was defined as a “transition-active neuron” if its actual similarity was greater than 99.95% of the distribution, representing a significantly higher possibility of a positive correlation between neuronal activity and speed transition.

### Behavioral testing

After 2 weeks of training on the variable speed wheel, we tested mice on the open field, two-tiered ladder, accelerating rotarod, elevated plus maze, and grip strength tests with age-matched littermates who had not been trained (naïve mice). For the open field test, a mouse was released into the center of the square open field arena and then allowed to move freely for 10 min without interruption or interference. A fast-speed camera on top of the arena captured a video of the mouse during the test. The videos were analyzed by software TopScan (CleverSys Inc., VA, USA).

A ladder walking behavior–analyzing system (CleverSys Inc., VA, USA) was used for the ladder test. This is a horizontal ladder composed of 2 × 37 rungs. The rungs are spaced 15 mm apart and alternate heights between high and low so that the mice prefer to walk on the high rungs. Steps on low rungs are considered missteps. Thus, fewer missteps mean better motor coordination. There is one shelter box at each end of the ladder. In the beginning of the test, one mouse is released into the left shelter box. Then, 5 s after the mouse leaves the box to walk on the ladder, a light is turned on in the shelter box. A fan is turned on after another 5 s to further encourage the mouse to walk on the ladder rather than return to the safety of the shelter. Walking from one end of the ladder to the other is defined as one trial. Each mouse performed 20 trials, during which we measured the time needed to cross the ladder and the number of correct steps (steps between adjacent high rungs) and irregular steps (steps from high rung to low rung, from low rung to low rung, crossing boundary to rungs on the other side, and backward steps).

The accelerating rotarod assay was performed as previously described (*74*). *Mecp2*-null mice and controls were placed on an accelerating rotarod apparatus (IITC Inc.) for four trials in 1 day with a 15-min rest interval between trials. Each trial lasted for a maximum of 5 min, during which the rod accelerated linearly from 4 to 40 rpm. For each trial, we recorded the latency to fall (i.e., the amount of time it took for each mouse to fall from the rod) and noted when the mice first stopped locomoting by holding the rotating rod.

The elevated plus maze assay was performed as previously described (*75*). The elevated plus maze apparatus consists of a plus-shaped platform that is elevated above the floor. Two opposite arms of the maze are walled, whereas the other two arms are open with a small threshold along the sides. Mice were placed in the center part of the maze facing one of the two open arms. The number of entries and the amount of time the animal spent in the open and closed arms were scored with TopScan behavioral data acquisition software (CleverSys, Reston, VA, USA).

For the grip strength test, each mouse was tested twice on two adjacent days by following the procedure as previously described (*76*). Each mouse was placed on a grid and exercised by pulling its tail without the mouse losing its grip. The action was repeated for 20 paw grips. The following five grips were measured using a dynamometer (Chatillon Digital Force Gauge, DFIS 2, Columbus Instruments, Columbus, OH). The maximal force applied to the dynamometer while on the grid was recorded. The maximum value of the two tests was used in fig. S12A. We also compared the mean value of 2 days of tests and got same results.

### Statistics

Linear mixed models were used to obtain *P* values. All response types are assumed to be Gaussian. Fixed effects in the linear regression model are given by (i) a factor variable that represents the trial number (i.e., day of the experiment) and (ii) the mouse type (i.e., WT or *Mecp2*-null mice). The presented *P* values are for the latter covariate. In addition, separate random effects for each mouse were fit, and an AR(1) correlation model was used to model the temporal correlation across the trials. We used R (R Core Team, 2019; www.R-project.org) and the R package “lmerTest” (*77*) or SPSS Version 26 (IBM Inc., Chicago, IL, USA) for fitting the models and performing inferences. In Fig. 3 (A and B), *P* values are obtained using Wilcoxon signed-rank test by SPSS.

## Notes

View/request a protocol for this paper from *Bio-protocol*.

## References

[1] R. M. Costa, D. Cohen, M. A. L. Nicolelis, Differential corticostriatal plasticity during fast and slow motor skill learning in mice. Curr. Biol. 14, 1124–1134 (2004). 15242609 10.1016/j.cub.2004.06.053

[2] T. Komiyama, T. R. Sato, D. H. O’Connor, Y.-X. Zhang, D. Huber, B. M. Hooks, M. Gabitto, K. Svoboda, Learning-related fine-scale specificity imaged in motor cortex circuits of behaving mice. Nature 464, 1182–1186 (2010). 20376005 10.1038/nature08897

[3] Y. Masamizu, Y. R. Tanaka, Y. H. Tanaka, R. Hira, F. Ohkubo, K. Kitamura, Y. Isomura, T. Okada, M. Matsuzaki, Two distinct layer-specific dynamics of cortical ensembles during learning of a motor task. Nat. Neurosci. 17, 987–994 (2014). 24880217 10.1038/nn.3739

[4] A. J. Peters, S. X. Chen, T. Komiyama, Emergence of reproducible spatiotemporal activity during motor learning. Nature 510, 263–267 (2014). 24805237 10.1038/nature13235

[5] J. Cichon, W. B. Gan, Branch-specific dendritic Ca^2+^ spikes cause persistent synaptic plasticity. Nature 520, 180–185 (2015). 25822789 10.1038/nature14251 PMC4476301

[6] M. Fu, X. Yu, J. Lu, Y. Zuo, Repetitive motor learning induces coordinated formation of clustered dendritic spines in vivo. Nature 483, 92–95 (2012). 22343892 10.1038/nature10844 PMC3292711

[7] G. Yang, F. Pan, W. B. Gan, Stably maintained dendritic spines are associated with lifelong memories. Nature 462, 920–924 (2009). 19946265 10.1038/nature08577 PMC4724802

[8] G. M. G. Shepherd, Corticostriatal connectivity and its role in disease. Nat. Rev. Neurosci. 14, 278–291 (2013). 23511908 10.1038/nrn3469 PMC4096337

[9] C. M. A. Pennartz, J. D. Berke, A. M. Graybiel, R. Ito, C. S. Lansink, M. Van Der Meer, A. D. Redish, K. S. Smith, P. Voorn, Corticostriatal interactions during learning, memory processing, and decision making. J. Neurosci. 29, 12831–12838 (2009). 19828796 10.1523/JNEUROSCI.3177-09.2009 PMC3849625

[10] R. E. Amir, I. B. Van Den Veyver, M. Wan, C. Q. Tran, U. Francke, H. Y. Zoghbi, Rett syndrome is caused by mutations in X-linked MECP2, encoding methyl-CpG-binding protein 2. Nat. Genet. 23, 185–188 (1999). 10508514 10.1038/13810

[11] A. J. Sandweiss, V. L. Brandt, H. Y. Zoghbi, Advances in understanding of Rett syndrome and MECP2 duplication syndrome: Prospects for future therapies. Lancet Neurol. 19, 689–698 (2020). 32702338 10.1016/S1474-4422(20)30217-9

[12] J. Guy, J. Gan, J. Selfridge, S. Cobb, A. Bird, Reversal of neurological defects in a mouse model of Rett syndrome. Science 315, 1143–1147 (2007). 17289941 10.1126/science.1138389 PMC7610836

[13] I. T. J. Wang, A. R. S. Reyes, Z. Zhou, Neuronal morphology in MeCP2 mouse models is intrinsically variable and depends on age, cell type, and Mecp2 mutation. Neurobiol. Dis. 58, 3–12 (2013). 23659895 10.1016/j.nbd.2013.04.020 PMC3748238

[14] T. Fukuda, M. Itoh, T. Ichikawa, K. Washiyama, Y. Goto, Delayed maturation of neuronal architecture and synaptogenesis in cerebral cortex of Mecp2-deficient mice. J. Neuropathol. Exp. Neurol. 64, 537–544 (2005). 15977646 10.1093/jnen/64.6.537

[15] T. Gemelli, O. Berton, E. D. Nelson, L. I. Perrotti, R. Jaenisch, L. M. Monteggia, Postnatal loss of methyl-CpG binding protein 2 in the forebrain is sufficient to mediate behavioral aspects of Rett syndrome in mice. Biol. Psychiatry 59, 468–476 (2006). 16199017 10.1016/j.biopsych.2005.07.025

[16] H. Cheval, J. Guy, C. Merusi, D. De Sousa, J. Selfridge, A. Bird, Postnatal inactivation reveals enhanced requirement for MeCP2 at distinct age windows. Hum. Mol. Genet. 21, 3806–3814 (2012). 22653753 10.1093/hmg/dds208 PMC3412380

[17] F. Du, M. V. C. Nguyen, A. Karten, C. A. Felice, G. Mandel, N. Ballas, Acute and crucial requirement for MeCP2 function upon transition from early to late adult stages of brain maturation. Hum. Mol. Genet. 25, 1690–1702 (2016). 26908602 10.1093/hmg/ddw038 PMC4986326

[18] S. Hao, B. Tang, Z. Wu, K. Ure, Y. Sun, H. Tao, Y. Gao, A. J. Patel, D. J. Curry, R. C. Samaco, H. Y. Zoghbi, J. Tang, Forniceal deep brain stimulation rescues hippocampal memory in Rett syndrome mice. Nature 526, 430–434 (2015). 26469053 10.1038/nature15694 PMC4828032

[19] H. Lu, R. T. Ash, L. He, S. E. Kee, W. Wang, D. Yu, S. Hao, X. Meng, K. Ure, A. Ito-Ishida, B. Tang, Y. Sun, D. Ji, J. Tang, B. R. Arenkiel, S. M. Smirnakis, H. Y. Zoghbi, Loss and gain of MeCP2 cause similar hippocampal circuit dysfunction that is rescued by deep brain stimulation in a Rett syndrome mouse model. Neuron 91, 739–747 (2016). 27499081 10.1016/j.neuron.2016.07.018 PMC5019177

[20] J. Guy, B. Hendrich, M. Holmes, J. E. Martin, A. Bird, A mouse Mecp2-null mutation causes neurological symptoms that mimic Rett syndrome. Nat. Genet. 27, 322–326 (2001). 11242117 10.1038/85899

[21] J. A. Hosp, A. Pekanovic, M. S. Rioult-Pedotti, A. R. Luft, Dopaminergic projections from midbrain to primary motor cortex mediate motor skill learning. J. Neurosci. 31, 2481–2487 (2011). 21325515 10.1523/JNEUROSCI.5411-10.2011 PMC6623715

[22] R. C. Samaco, C. Mandel-Brehm, H. T. Chao, C. S. Ward, S. L. Fyffe-Maricich, J. Ren, K. Hyland, C. Thaller, S. M. Maricich, P. Humphreys, J. J. Greer, A. Percy, D. G. Glaze, H. Y. Zoghbi, J. L. Neul, Loss of MeCP2 in aminergic neurons causes cell-autonomous defects in neurotransmitter synthesis and specific behavioral abnormalities. Proc. Natl. Acad. Sci. U.S.A. 106, 21966–21971 (2009). 20007372 10.1073/pnas.0912257106 PMC2799790

[23] P. D. Ross, J. Guy, J. Selfridge, B. Kamal, N. Bahey, K. Elizabeth Tanner, T. H. Gillingwater, R. A. Jones, C. M. Loughrey, C. S. McCarroll, M. E. S. Bailey, A. Bird, S. Cobb, Exclusive expression of MeCP2 in the nervous system distinguishes between brain and peripheral Rett syndrome-like phenotypes. Hum. Mol. Genet. 25, 4389–4404 (2016). 28173151 10.1093/hmg/ddw269 PMC5886038

[24] A. Mathis, P. Mamidanna, K. M. Cury, T. Abe, V. N. Murthy, M. W. Mathis, M. Bethge, DeepLabCut: Markerless pose estimation of user-defined body parts with deep learning. Nat. Neurosci. 21, 1281–1289 (2018). 30127430 10.1038/s41593-018-0209-y

[25] X. Meng, W. Wang, H. Lu, L.-J. He, W. Chen, E. S. Chao, M. L. Fiorotto, B. Tang, J. A. Herrera, M. L. Seymour, J. L. Neul, F. A. Pereira, J. Tang, M. Xue, H. Y. Zoghbi, Manipulations of MeCP2 in glutamatergic neurons highlight their contributions to Rett and other neurological disorders. eLife 5, e14199 (2016). 27328325 10.7554/eLife.14199 PMC4946906

[26] I. H. Jenkins, D. J. Brooks, P. D. Nixon, R. S. J. Frackowiak, R. E. Passingham, Motor sequence learning: A study with positron emission tomography. J. Neurosci. 14, 3775–3790 (1994). 8207487 10.1523/JNEUROSCI.14-06-03775.1994 PMC6576955

[27] L. Ma, B. Wang, S. Narayana, E. Hazeltine, X. Chen, D. A. Robin, P. T. Fox, J. Xiong, Changes in regional activity are accompanied with changes in inter-regional connectivity during 4 weeks motor learning. Brain Res. 1318, 64–76 (2010). 20051230 10.1016/j.brainres.2009.12.073 PMC2826520

[28] N. Picard, Y. Matsuzaka, P. L. Strick, Extended practice of a motor skill is associated with reduced metabolic activity in M1. Nat. Neurosci. 16, 1340–1347 (2013). 23912947 10.1038/nn.3477 PMC3757119

[29] N. F. Wymbs, S. T. Grafton, The human motor system supports sequence-specific representations over multiple training-dependent timescales. Cereb. Cortex 25, 4213–4225 (2015). 24969473 10.1093/cercor/bhu144 PMC4747644

[30] T. Taillefumier, M. O. Magnasco, A phase transition in the first passage of a Brownian process through a fluctuating boundary with implications for neural coding. Proc. Natl. Acad. Sci. U.S.A. 110, E1438–E1443 (2013). 23536302 10.1073/pnas.1212479110 PMC3631650

[31] N. A. Frost, A. Haggart, V. S. Sohal, Dynamic patterns of correlated activity in the prefrontal cortex encode information about social behavior. PLoS Biol. 19, e3001235 (2021). 33939689 10.1371/journal.pbio.3001235 PMC8118626

[32] J. Schäfer, K. Strimmer, A shrinkage approach to large-scale covariance matrix estimation and implications for functional genomics. Stat. Appl. Genet. Mol. Biol. 4, Article32 (2005). 16646851 10.2202/1544-6115.1175

[33] R. R. Stein, D. S. Marks, C. Sander, Inferring pairwise interactions from biological data using maximum-entropy probability models. PLoS Comput. Biol. 11, e1004182 (2015). 26225866 10.1371/journal.pcbi.1004182 PMC4520494

[34] H. Toh, K. Horimoto, Inference of a genetic network by a combined approach of cluster analysis and graphical Gaussian modeling. Bioinformatics 18, 287–297 (2002). 11847076 10.1093/bioinformatics/18.2.287

[35] L. Wood, N. W. Gray, Z. Zhou, M. E. Greenberg, G. M. G. Shepherd, Synaptic circuit abnormalities of motor-frontal layer 2/3 pyramidal neurons in an RNA interference model of methyl-CpG-binding protein 2 deficiency. J. Neurosci. 29, 12440–12448 (2009). 19812320 10.1523/JNEUROSCI.3321-09.2009 PMC2782478

[36] V. S. Dani, S. B. Nelson, Intact long-term potentiation but reduced connectivity between neocortical layer 5 pyramidal neurons in a mouse model of Rett syndrome. J. Neurosci. 29, 11263–11270 (2009). 19741133 10.1523/JNEUROSCI.1019-09.2009 PMC2765053

[37] B. Kriener, M. Helias, A. Aertsen, S. Rotter, Correlations in spiking neuronal networks with distance dependent connections. J. Comput. Neurosci. 27, 177–200 (2009). 19568923 10.1007/s10827-008-0135-1 PMC2731936

[38] M. R. Cohen, A. Kohn, Measuring and interpreting neuronal correlations. Nat. Neurosci. 14, 811–819 (2011). 21709677 10.1038/nn.2842 PMC3586814

[39] M. Schenkman, C. G. Moore, W. M. Kohrt, D. A. Hall, A. Delitto, C. L. Comella, D. A. Josbeno, C. L. Christiansen, B. D. Berman, B. M. Kluger, E. L. Melanson, S. Jain, J. A. Robichaud, C. Poon, D. M. Corcos, Effect of high-intensity treadmill exercise on motor symptoms in patients with de novo Parkinson disease a phase 2 randomized clinical trial. JAMA Neurol. 75, 219–226 (2018). 29228079 10.1001/jamaneurol.2017.3517 PMC5838616

[40] E. B. Beall, M. J. Lowe, J. L. Alberts, A. M. M. Frankemolle, A. K. Thota, C. Shah, M. D. Phillips, The effect of forced-exercise therapy for Parkinson’s disease on motor cortex functional connectivity. Brain Connect. 3, 190–198 (2013). 23316956 10.1089/brain.2012.0104 PMC3634143

[41] C. Petrus, S. R. Adamson, L. Block, S. J. Einarson, M. Sharifnejad, S. R. Harris, Effects of exercise interventions on stereotypic behaviours in children with autism spectrum disorder. Physiother. Can. 60, 134–145 (2008). 20145777 10.3138/physio.60.2.134 PMC2792819

[42] N. A. Stearns, L. R. Schaevitz, H. Bowling, N. Nag, U. V. Berger, J. Berger-Sweeney, Behavioral and anatomical abnormalities in Mecp2 mutant mice: A model for Rett syndrome. Neuroscience 146, 907–921 (2007). 17383101 10.1016/j.neuroscience.2007.02.009

[43] J. Downs, J. Rodger, C. Li, X. Tan, N. Hu, K. Wong, N. De Klerk, H. Leonard, Environmental enrichment intervention for Rett syndrome: An individually randomised stepped wedge trial. Orphanet J. Rare Dis. 13, 3 (2018). 29321033 10.1186/s13023-017-0752-8 PMC5764021

[44] M. Kondo, L. J. Gray, G. J. Pelka, J. Christodoulou, P. P. L. Tam, A. J. Hannan, Environmental enrichment ameliorates a motor coordination deficit in a mouse model of Rett syndrome—*Mecp2* gene dosage effects and BDNF expression. Eur. J. Neurosci. 27, 3342–3350 (2008). 18557922 10.1111/j.1460-9568.2008.06305.x

[45] G. Lonetti, A. Angelucci, L. Morando, E. M. Boggio, M. Giustetto, T. Pizzorusso, Early environmental enrichment moderates the behavioral and synaptic phenotype of MeCP2 null mice. Biol. Psychiatry 67, 657–665 (2010). 20172507 10.1016/j.biopsych.2009.12.022

[46] A. J. Peters, H. Liu, T. Komiyama, Learning in the rodent motor cortex. Annu. Rev. Neurosci. 40, 77–97 (2017). 28375768 10.1146/annurev-neuro-072116-031407 PMC5714655

[47] A. F. Teich, N. Qian, Learning and adaptation in a recurrent model of V1 orientation selectivity. J. Neurophysiol. 89, 2086–2100 (2003). 12611961 10.1152/jn.00970.2002

[48] H. Makino, E. J. Hwang, N. G. Hedrick, T. Komiyama, Circuit mechanisms of sensorimotor learning. Neuron 92, 705–721 (2016). 27883902 10.1016/j.neuron.2016.10.029 PMC5131723

[49] K. Ganguly, J. M. Carmena, Emergence of a stable cortical map for neuroprosthetic control. PLoS Biol. 7, e1000153 (2009). 19621062 10.1371/journal.pbio.1000153 PMC2702684

[50] K. Ganguly, J. M. Carmena, Neural correlates of skill acquisition with a cortical brain-machine interface. J. Mot. Behav. 42, 355–360 (2010). 21184353 10.1080/00222895.2010.526457

[51] V. R. Athalye, J. M. Carmena, R. M. Costa, Neural reinforcement: Re-entering and refining neural dynamics leading to desirable outcomes. Curr. Opin. Neurobiol. 60, 145–154 (2020). 31877493 10.1016/j.conb.2019.11.023

[52] B. M. Hooks, T. Mao, D. A. Gutnisky, N. Yamawaki, K. Svoboda, G. M. G. Shepherd, Organization of cortical and thalamic input to pyramidal neurons in mouse motor cortex. J. Neurosci. 33, 748–760 (2013). 23303952 10.1523/JNEUROSCI.4338-12.2013 PMC3710148

[53] S. Levy, M. Lavzin, H. Benisty, A. Ghanayim, U. Dubin, S. Achvat, Z. Brosh, F. Aeed, B. D. Mensh, Y. Schiller, R. Meir, O. Barak, R. Talmon, A. W. Hantman, J. Schiller, Cell-type-specific outcome representation in the primary motor cortex. Neuron 107, 954–971.e9 (2020). 32589878 10.1016/j.neuron.2020.06.006

[54] B. P. Jung, D. G. M. Jugloff, G. Zhang, R. Logan, S. Brown, J. H. Eubanks, The expression of methyl CpG binding factor MeCP2 correlates with cellular differentiation in the developing rat brain and in cultured cells. J. Neurobiol. 55, 86–96 (2003). 12605461 10.1002/neu.10201

[55] L. Rietveld, D. P. Stuss, D. McPhee, K. R. Delaney, Genotype-specific effects of Mecp2 loss-of-function on morphology of layer V pyramidal neurons in heterozygous female Rett syndrome model mice. Front. Cell. Neurosci. 9, 145 (2015). 25941473 10.3389/fncel.2015.00145 PMC4403522

[56] R. Z. Chen, S. Akbarian, M. Tudor, R. Jaenisch, Deficiency of methyl-CpG binding protein-2 in CNS neurons results in a Rett-like phenotype in mice. Nat. Genet. 27, 327–331 (2001). 11242118 10.1038/85906

[57] J. Noutel, Y. K. Hong, B. Leu, E. Kang, C. Chen, Experience-dependent retinogeniculate synapse remodeling is abnormal in MeCP2-deficient mice. Neuron 70, 35–42 (2011). 21482354 10.1016/j.neuron.2011.03.001 PMC3082316

[58] N. P. Achilly, W. Wang, H. Y. Zoghbi, Presymptomatic training mitigates functional deficits in a mouse model of Rett syndrome. Nature 592, 596–600 (2021). 33762729 10.1038/s41586-021-03369-7 PMC8093094

[59] T. Y. C. Pang, N. C. Stam, J. Nithianantharajah, M. L. Howard, A. J. Hannan, Differential effects of voluntary physical exercise on behavioral and brain-derived neurotrophic factor expression deficits in Huntington’s disease transgenic mice. Neuroscience 141, 569–584 (2006). 16716524 10.1016/j.neuroscience.2006.04.013

[60] M. Valentin-Gudiol, C. Bagur-Calafat, M. Girabent-Farrés, M. Hadders-Algra, K. Mattern-Baxter, R. Angulo-Barroso, Treadmill interventions with partial body weight support in children under six years of age at risk of neuromotor delay: A report of a Cochrane systematic review and meta-analysis. Eur. J. Phys. Rehabil. Med. 49, 67–91 (2013). 23575201

[61] G. Larsson, P. O. O. Julu, I. Witt Engerström, M. Sandlund, B. Lindström, Walking on treadmill with Rett syndrome—Effects on the autonomic nervous system. Res. Dev. Disabil. 83, 99–107 (2018). 30193160 10.1016/j.ridd.2018.08.010

[62] L. Escobar Torres, M. E. Sanders, C. Belenguer Benitez, A. Melendez Ortega, Efficacy of an aquatic exercise program for 3 cases of Rett syndrome. Pediatr. Phys. Ther. 31, E6–E13 (2019). 10.1097/PEP.0000000000000655 31568388

[63] M. Stahlhut, J. Downs, K. Wong, A. M. Bisgaard, E. Nordmark, Feasibility and effectiveness of an individualized 12-week “uptime” participation (U-PART) intervention in girls and women with Rett syndrome. Phys. Ther. 100, 168–179 (2020). 31584667 10.1093/ptj/pzz138

[64] T. Imamura, T. Nakayama, J. Nakayama, N. Iwasaki, A patient with Rett syndrome maintained motor function by periodic rehabilitation therapy and proactive daily activities. Prog. Rehabil. Med. 5, 20200014 (2020). 32844127 10.2490/prm.20200014 PMC7429559

[65] M. A. Kondo, L. J. Gray, G. J. Pelka, S. K. Leang, J. Christodoulou, P. P. L. Tam, A. J. Hannan, Affective dysfunction in a mouse model of Rett syndrome: Therapeutic effects of environmental stimulation and physical activity. Dev. Neurobiol. 76, 209–224 (2016). 26019053 10.1002/dneu.22308

[66] B. Kerr, P. A. Silva, K. Walz, J. I. Young, Unconventional transcriptional response to environmental enrichment in a mouse model of Rett syndrome. PLOS ONE 5, e11534 (2010). 20634955 10.1371/journal.pone.0011534 PMC2902516

[67] R. T. Ash, P. G. Fahey, J. Park, H. Y. Zoghbi, S. M. Smirnakis, Increased axonal bouton stability during learning in the mouse model of MECP2 duplication syndrome. eNeuro. 5, ENEURO.0056-17.2018 (2018). 10.1523/ENEURO.0056-17.2018 PMC6086213 30105297

[68] E. A. Pnevmatikakis, D. Soudry, Y. Gao, T. A. Machado, J. Merel, D. Pfau, T. Reardon, Y. Mu, C. Lacefield, W. Yang, M. Ahrens, R. Bruno, T. M. Jessell, D. S. Peterka, R. Yuste, L. Paninski, Simultaneous denoising, deconvolution, and demixing of calcium imaging data. Neuron 89, P285–P299 (2016). 10.1016/j.neuron.2015.11.037 PMC4881387 26774160

[69] A. Giovannucci, J. Friedrich, P. Gunn, J. Kalfon, B. L. Brown, S. A. Koay, J. Taxidis, F. Najafi, J. L. Gauthier, P. Zhou, B. S. Khakh, D. W. Tank, D. B. Chklovskii, E. A. Pnevmatikakis, CaImAn an open source tool for scalable calcium imaging data analysis. eLife 8, e38173 (2019). 30652683 10.7554/eLife.38173 PMC6342523

[70] J. Mairal, F. Bach, J. Ponce, G. Sapiro, Online dictionary learning for sparse coding. ACM Int. Conf. Proc. Ser. 382, 689–696 (2009).

[71] J. T. Vogelstein, A. M. Packer, T. A. Machado, T. Sippy, B. Babadi, R. Yuste, L. Paninski, Fast nonnegative deconvolution for spike train inference from population calcium imaging. J. Neurophysiol. 104, 3691–3704 (2010). 20554834 10.1152/jn.01073.2009 PMC3007657

[72] J. Friedrich, P. Zhou, L. Paninski, Fast online deconvolution of calcium imaging data. PLoS Comput. Biol. 13, e1005423 (2017). 28291787 10.1371/journal.pcbi.1005423 PMC5370160

[73] E. A. Pnevmatikakis, A. Giovannucci, NoRMCorre: An online algorithm for piecewise rigid motion correction of calcium imaging data. J. Neurosci. Methods 291, 83–94 (2017). 28782629 10.1016/j.jneumeth.2017.07.031

[74] M. D. Shahbazian, J. I. Young, L. A. Yuva-Paylor, C. M. Spencer, B. A. Antalffy, J. L. Noebels, D. L. Armstrong, R. Paylor, H. Y. Zoghbi, Mice with truncated MeCP2 recapitulate many Rett syndrome features and display hyperacetylation of histone H3. Neuron 35, 243–254 (2002). 12160743 10.1016/s0896-6273(02)00768-7

[75] A. Holmes, Targeted gene mutation approaches to the study of anxiety-like behavior in mice. Neurosci. Biobehav. Rev. 25, 261–273 (2001). 11378180 10.1016/s0149-7634(01)00012-4

[76] E. Tuzun, S. Berrih-Aknin, T. Brenner, L. L. Kusner, R. Le Panse, H. Yang, S. Tzartos, P. Christadoss, Guidelines for standard preclinical experiments in the mouse model of myasthenia gravis induced by acetylcholine receptor immunization. Exp. Neurol. 270, 11–17 (2015). 25697844 10.1016/j.expneurol.2015.02.009

[77] A. Kuznetsova, P. B. Brockhoff, R. H. B. Christensen, lmerTest Package: Tests in linear mixed effects models. J. Stat. Softw. 82, 26 (2017).

