# Supplementary material for: Motor training improves coordination and anxiety in symptomatic Mecp2-null mice despite impaired functional connectivity within the motor circuit
Source: Sci Adv. 2021 Oct 22;7(43):eabf7467. doi: 10.1126/sciadv.abf7467 (PMC8535852; doi:10.1126/sciadv.abf7467)
Supplement: Supplementary file 1 — Figs. S1 to S13 Table S1 Legends for movies S1 to S4 [file sciadv.abf7467_sm.pdf]

Science Advances
AAAS

Supplementary Materials for

Motor training improves coordination and anxiety in symptomatic *Mecp2*-null mice despite impaired functional connectivity within the motor circuit

Yuanlei Yue, Pan Xu, Zhichao Liu, Xiaoqian Sun, Juntao Su, Hongfei Du, Lingling Chen, Ryan T. Ash, Stelios Smirnakis, Rahul Simha, Linda Kusner, Chen Zeng, Hui Lu*

*Corresponding author. Email: huilu@email.gwu.edu

Published 22 October 2021, *Sci. Adv.* **7**, eabf7467 (2021)
DOI: 10.1126/sciadv.abf7467

The PDF file includes:

Figs. S1 to S13
Table S1
Legends for movies S1 to S4

Other Supplementary Material for this manuscript includes the following:

Movies S1 to S4

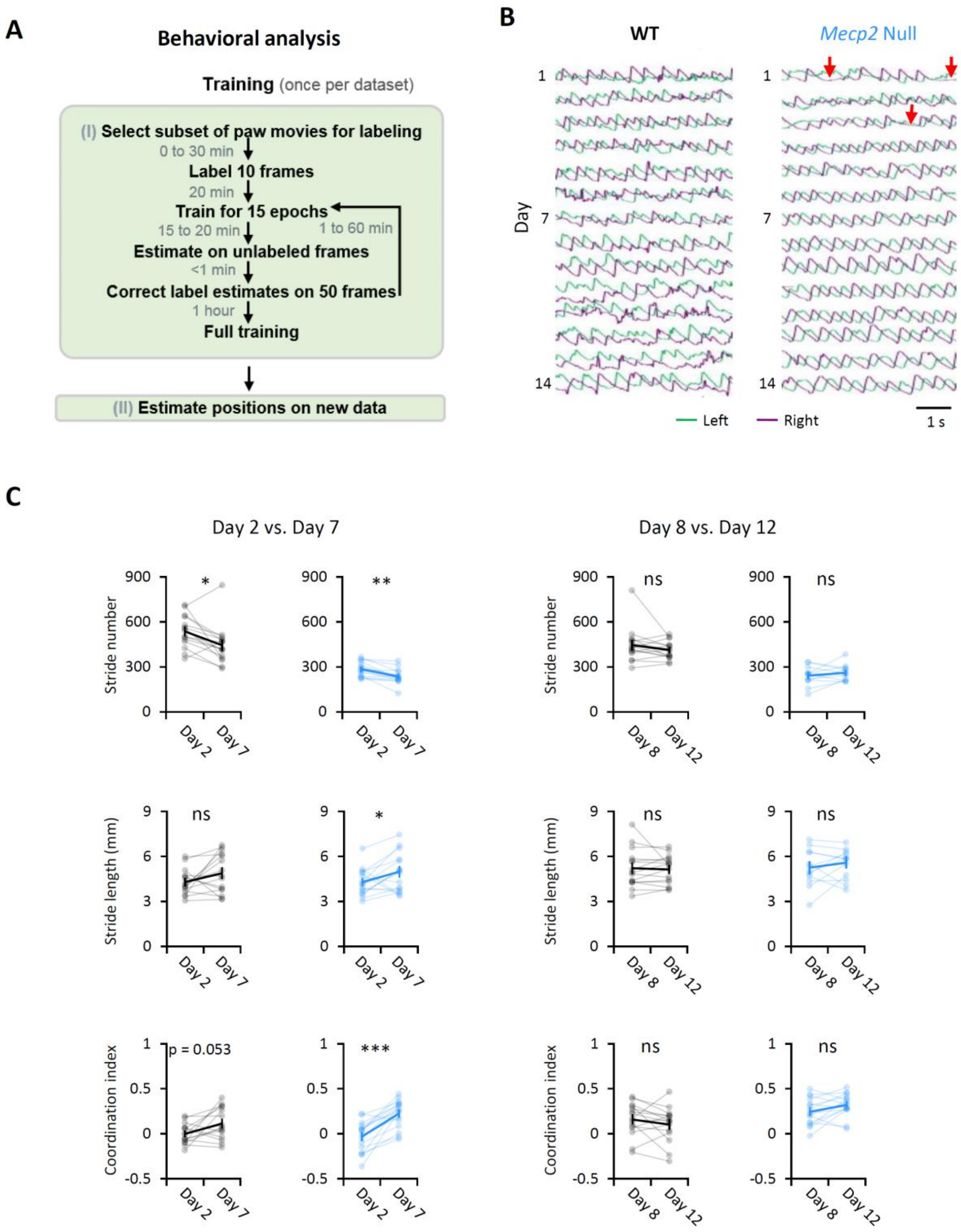

Fig. S1. Analysis of forepaw movement during running and degree of motor improvement during the first week vs. second week of training. (**A**) The workflow of a machine learning approach, DeepLabCut method, to track forepaw location. (**B**) Raw traces of forepaw locations across 14 days at speed 60 mm/s from one sample mouse in each group. (**C**) Comparison between changes in stride and coordination during the first and second week of training show greater improvement during the learning phase than the consolidation phase. WT: n=12 mice; *Mecp2*-null, n=14 mice. ns, not significant, * $P < 0.05$, ** $P < 0.01$, Wilcoxon signed-rank test.

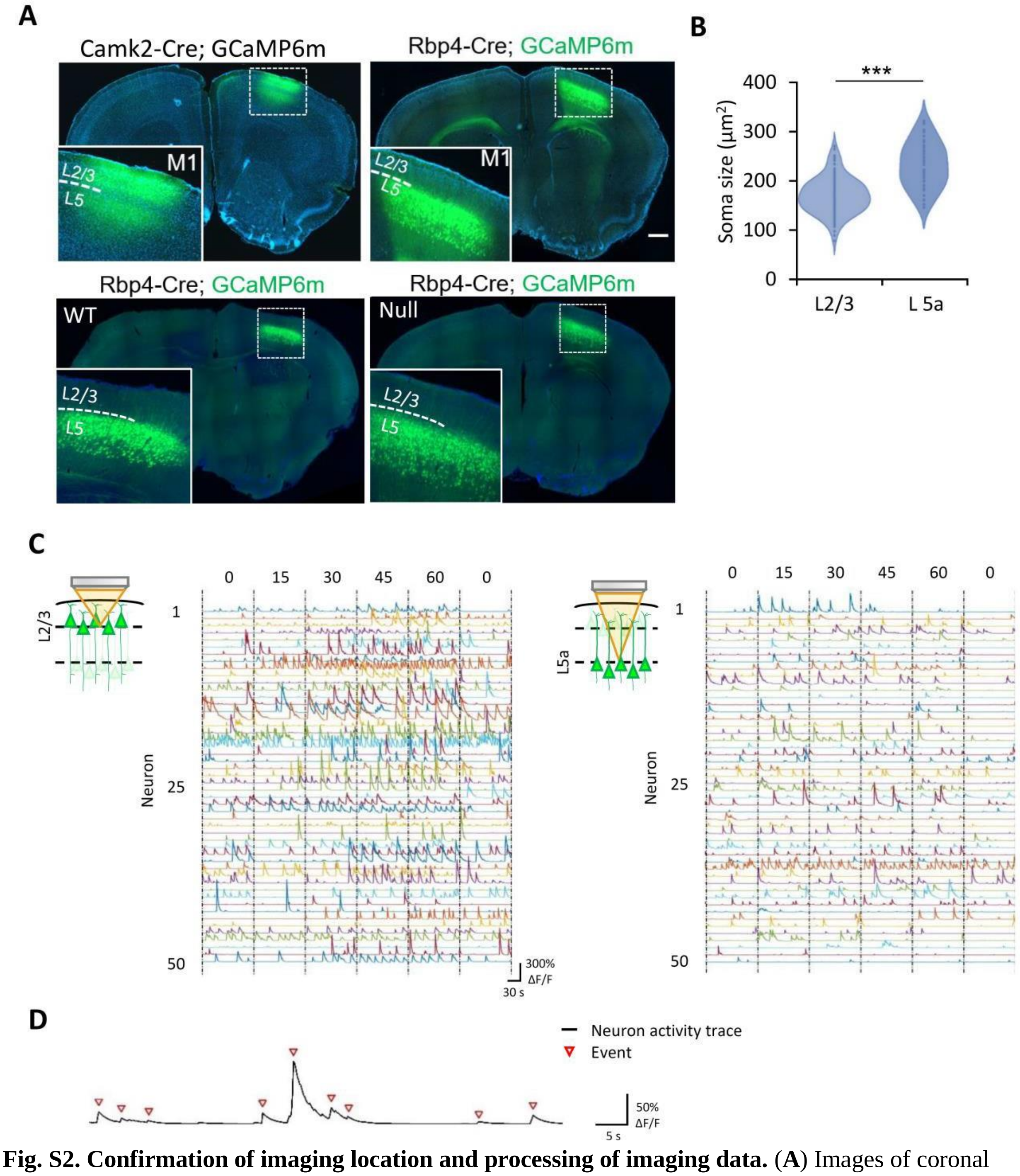

Fig. S2. Confirmation of imaging location and processing of imaging data. (**A**) Images of coronal sections showing expression of GCaMP6m in the area M1. Magnification of the boxed area is shown in the lower left corner of the larger images. Top, Rbp4-Cre-induced expression confirms the depth of L5a isaround 400-450 µm. Bottom, no noticeable difference of the depth of L5a between the WT and *Mecp2*- null mouse. Scale: 500 µm. (**B**) Violin plot with the addition of a rotated kernel density plot on each side comparing the soma size of detected neurons in L2/3 and L5a. *** $P < 0.001$ (Rank sum test) (**C**) Pre- processed sample traces of calcium dynamics in 50 neurons in L2/3 (left) and L5a (right) of one mouse during increase-speed mode. (**D**) Denoised neuronal activity trace; red triangles point to events.

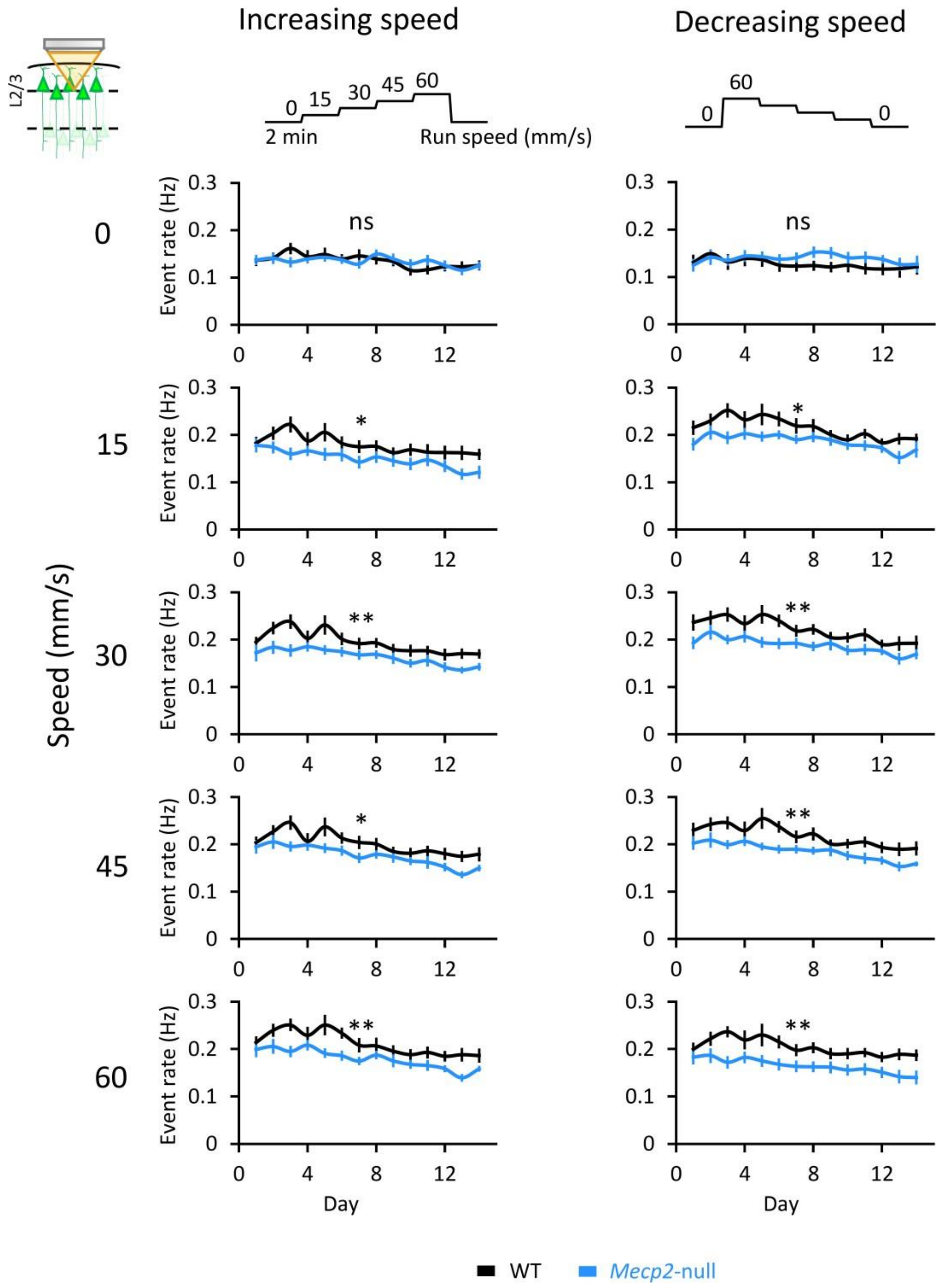

Fig. S3. Comparison of firing rates at different speeds in M1 area L2/3. Summary of event rates of WT and null neurons in L2/3 in session 1 (left, speeding-up mode) and session 2 (right, slowing-down mode) at each speed. WT: n=13 mice; Null, n=14 mice. Error bars represent mean ± SE. ns, not significant; * $P < 0.05$; ** $P < 0.01$, RM-ANOVA test.

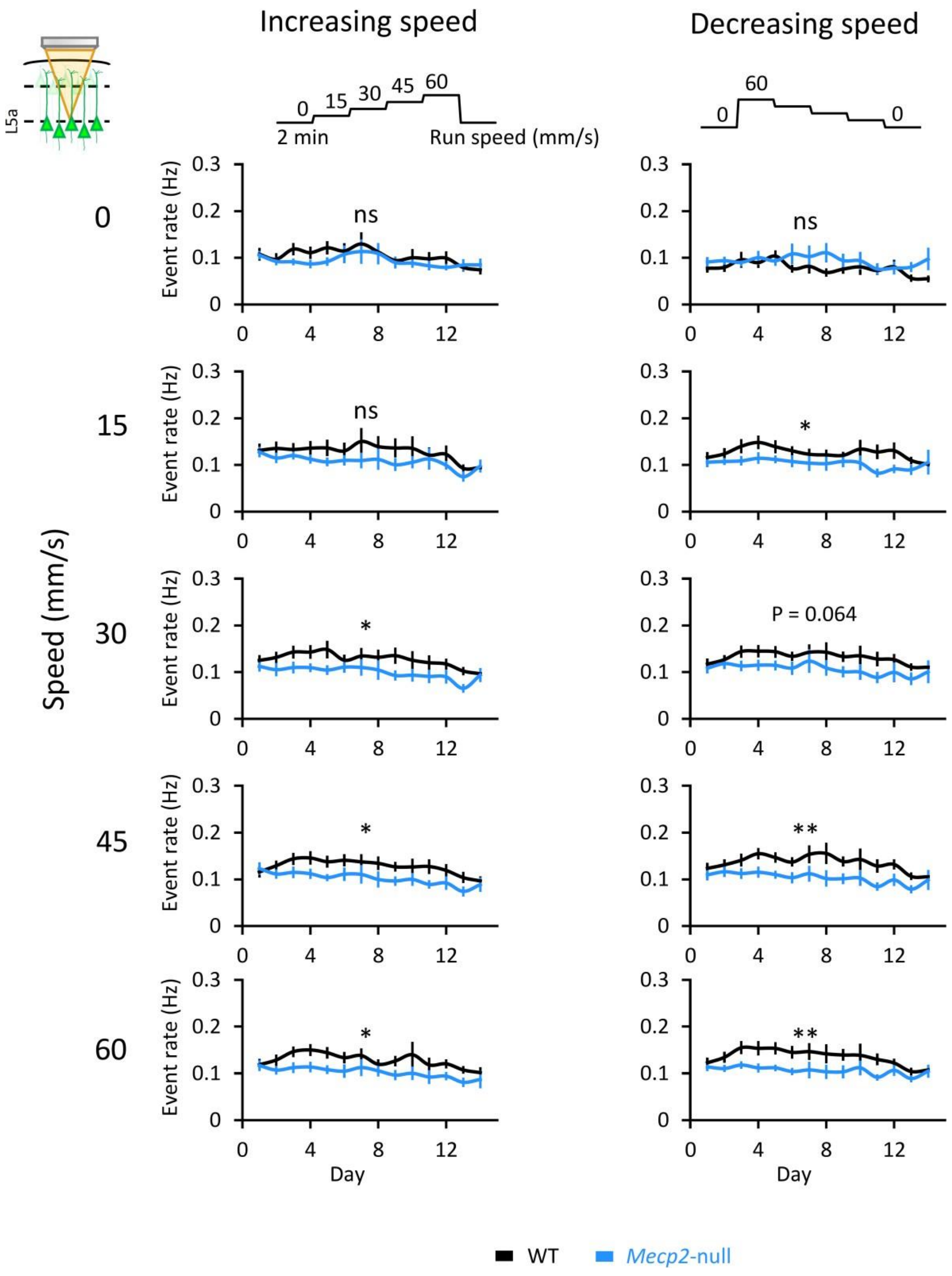

Fig. S4. Comparison of L5a firing rates at different speeds. Summary of event rates of WT and Nullneurons in L5a in session 1 (left, speeding-up mode) and session 2 (right, slowing-down mode) at each speed. WT: n=11 mice; Null, n=13 mice. Error bars represent mean ± SE. * $P < 0.05$; ** $P < 0.01$, RM-ANOVA test.

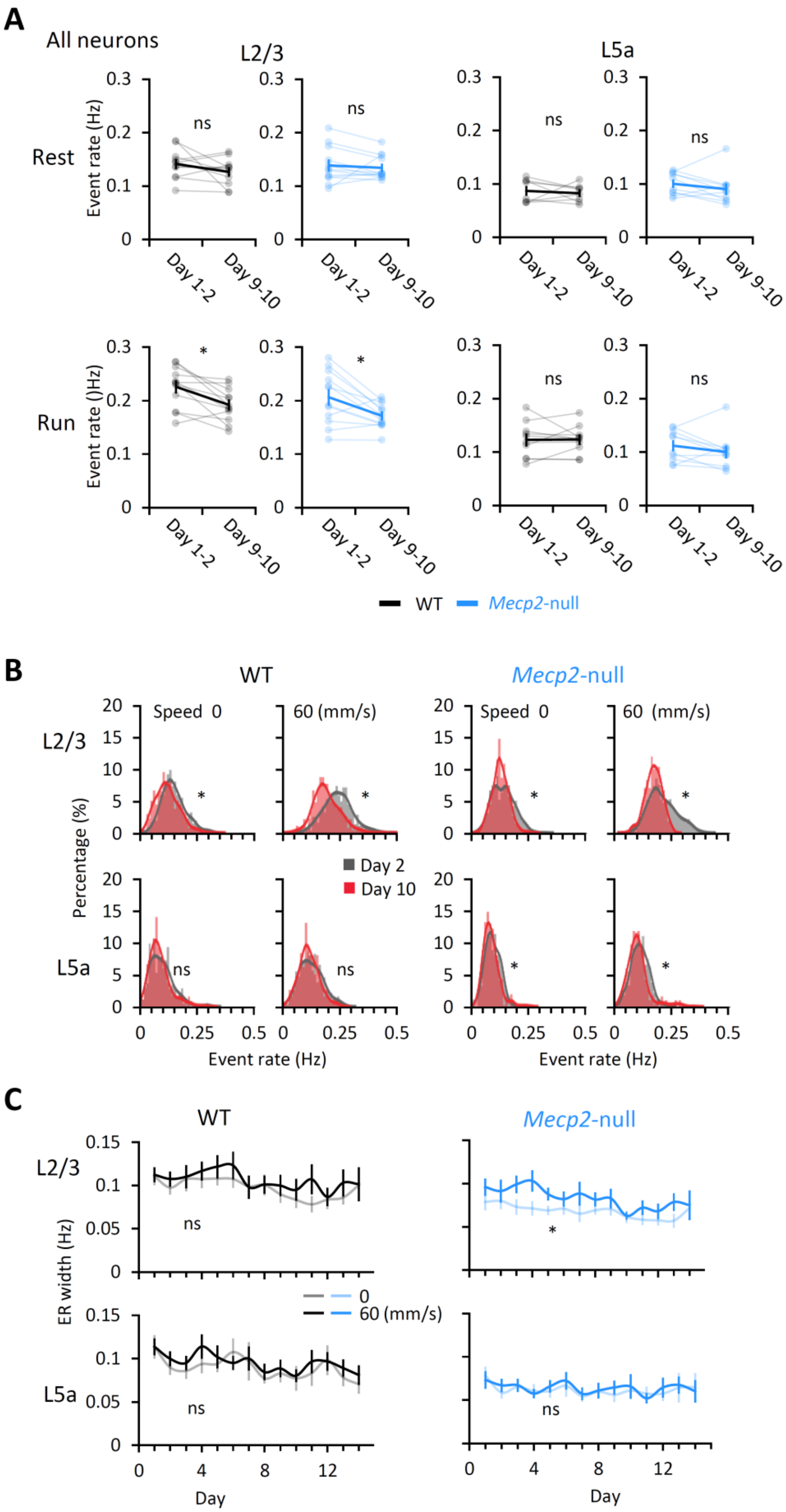
A
All neurons
L2/3
L5a
Rest
Run
Event rate (Hz)
Event rate ()Hz)
ns
*
Day 1-2
Day 9-10
WT
Mecp2-null
B
WT
Mecp2-null
Speed 0
60 (mm/s)
L2/3
L5a
Percentage (%)
Day 2
Day 10
Event rate (Hz)
C
WT
Mecp2-null
L2/3
L5a
ER width (Hz)
0
60 (mm/s)
Day

Fig. S5. Evolution of event rates over the course of learning. (**A**) Mean event rates of neurons in L2/3 and L5a during rest and running at 60 mm/sec block. Light lines connect data from an individual mouse. Dark lines connect the averaged data from all mice. L2/3 WT: n=11 mice; L2/3 *Mecp2*-null, n=12 mice. L5a WT: n=8 mice; L5a *Mecp2*-null, n=10 mice. Error bars represent mean ± SE. ns, not significant, * $P < 0.05$, Wilcoxon signed-rank test. (**B**) Distribution of neuronal event rates of all mice on days 2 and 10 . Motor learning narrows the distribution and lowers the rates over time. * $P < 0.0001$, Kolmogorov–Smirnov test. (**C**) The width of the event rate (ER) distribution in **B**, calculated as full width at half maximum of the amplitude of the distribution curve. L2/3 WT: n=13; L2/3 *Mecp2*-null: n=14; L5a WT: n=11; L5a *Mecp2*-null: n=13. * $P < 0.05$, ** $P < 0.01$, RM-ANOVA test.

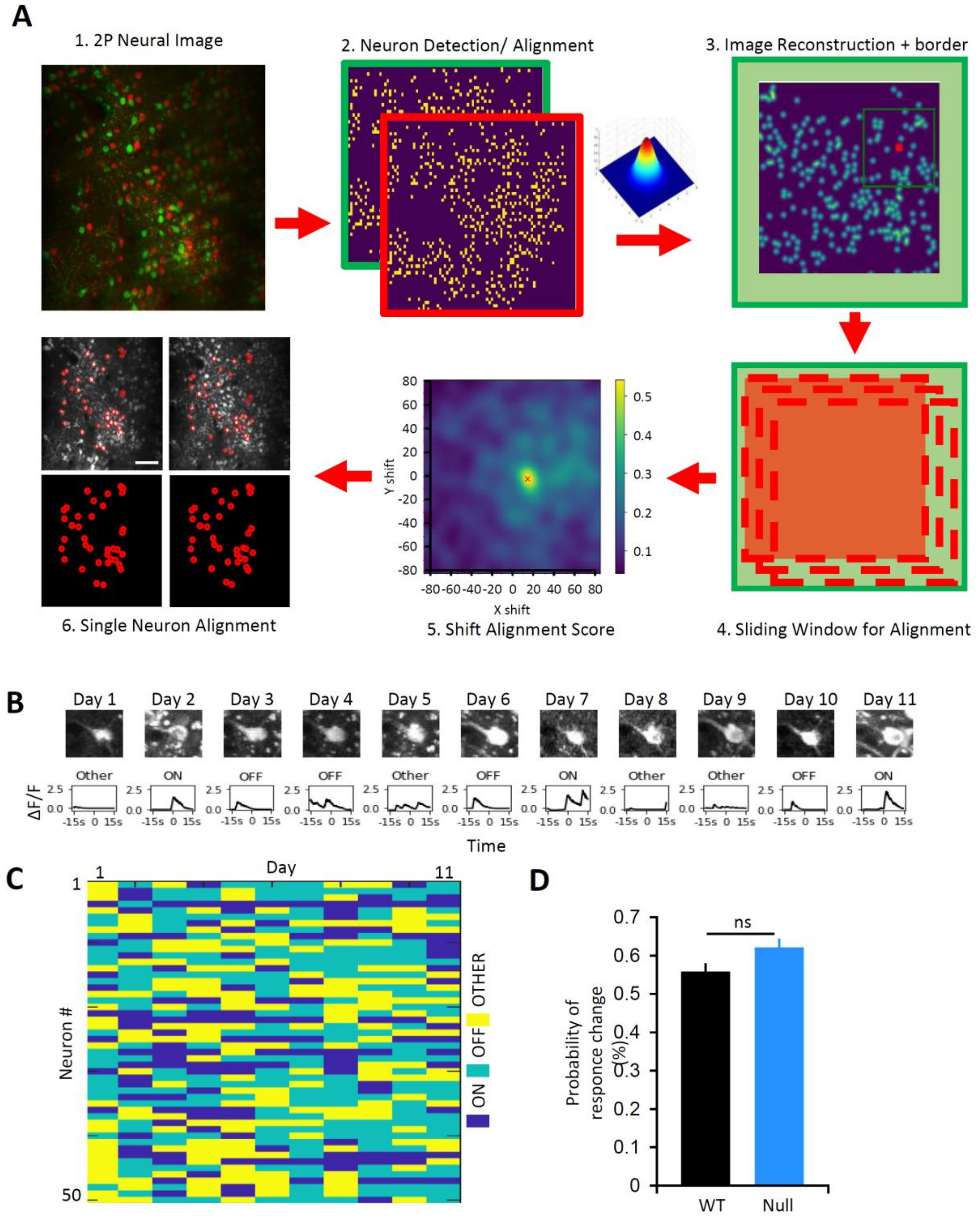

Fig. S6. Behavior of one neuron over eleven days. (**A**) Schematic of Global Alignment method (see Methods). About 20% of neurons can be followed over 12 days. (**B**) A representative neuron that showed different responses to the transition from rest to 15 mm/s (bottom row) over 11 different days. (**C**) The responses of 50 individual neurons to a transition from rest to 15 mm/s on each day over 11days. (**D**) Probability of response type changes between two adjacent days in WT and *Mecp2*-null mice. WT, n=7 mice; *Mecp2*-null, n=5 mice. ns, not significant, Two-tailed t-test.

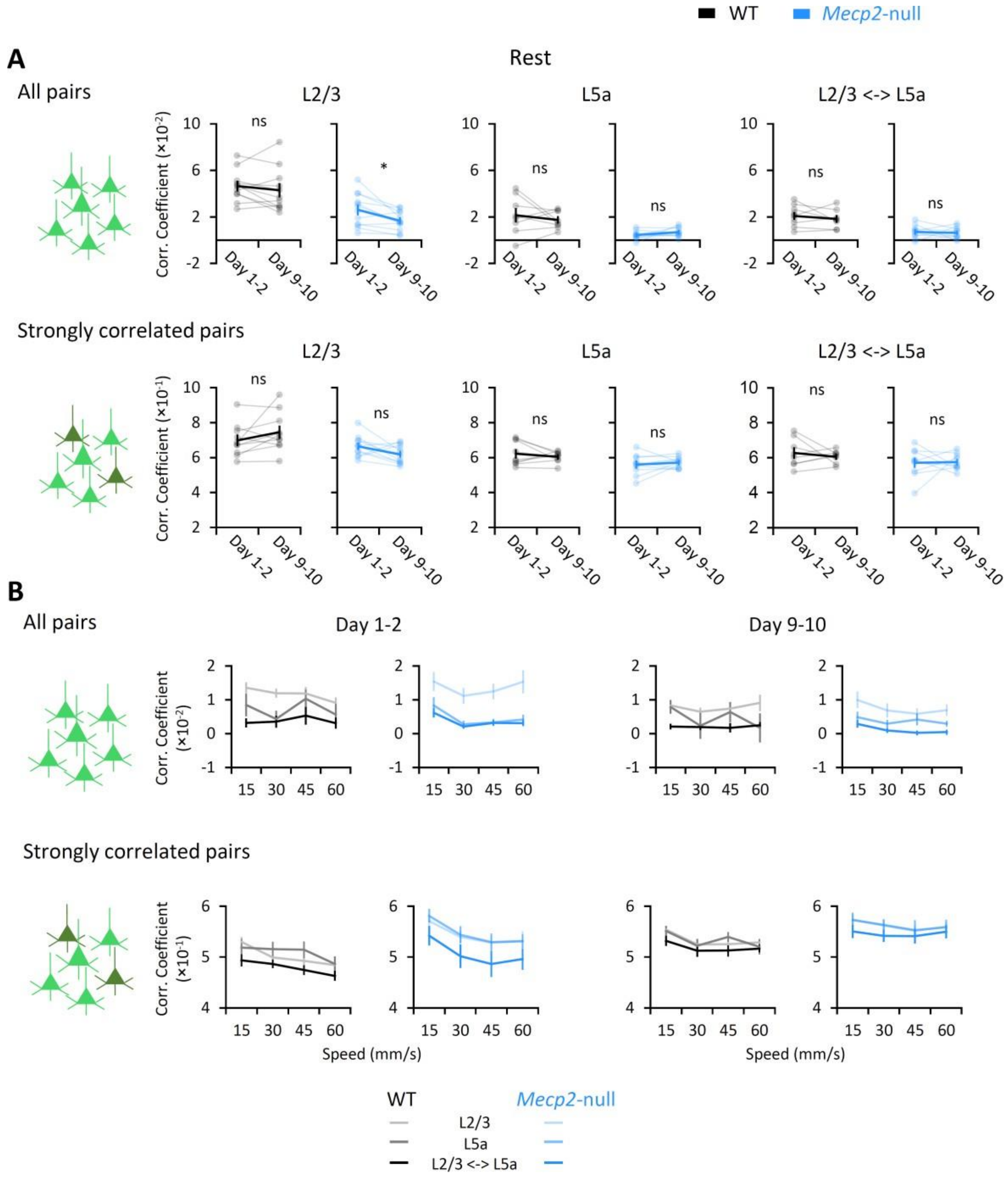

Fig. S7. Evolution of functional connectivity at rest and running. (**A**) Mean Pearson correlation coefficient (PCC) between pairs of neurons within L2/3, within L5a, and across both layers during rest (top: all pairs of correlation; bottom: strongly correlated pairs, defined by a PCC > twice the standard deviation). Light lines connect data from an individual mouse; dark lines show the average from all mice. L2/3 WT, n=11 mice; L2/3 *Mecp2*-null, n=12 mice; L5a WT,n=8 mice; L5a *Mecp2*-null, n=10 mice. Across both layers, WT, n=8 mice; *Mecp2*-null, n=10 mice, Error bars represent mean ± SE. * $P < 0.05$, Wilcoxon signed-rank test. (**B**) Average PCC at each speed from all the mice of each group (top: all pairs; bottom: strongly correlated pairs).

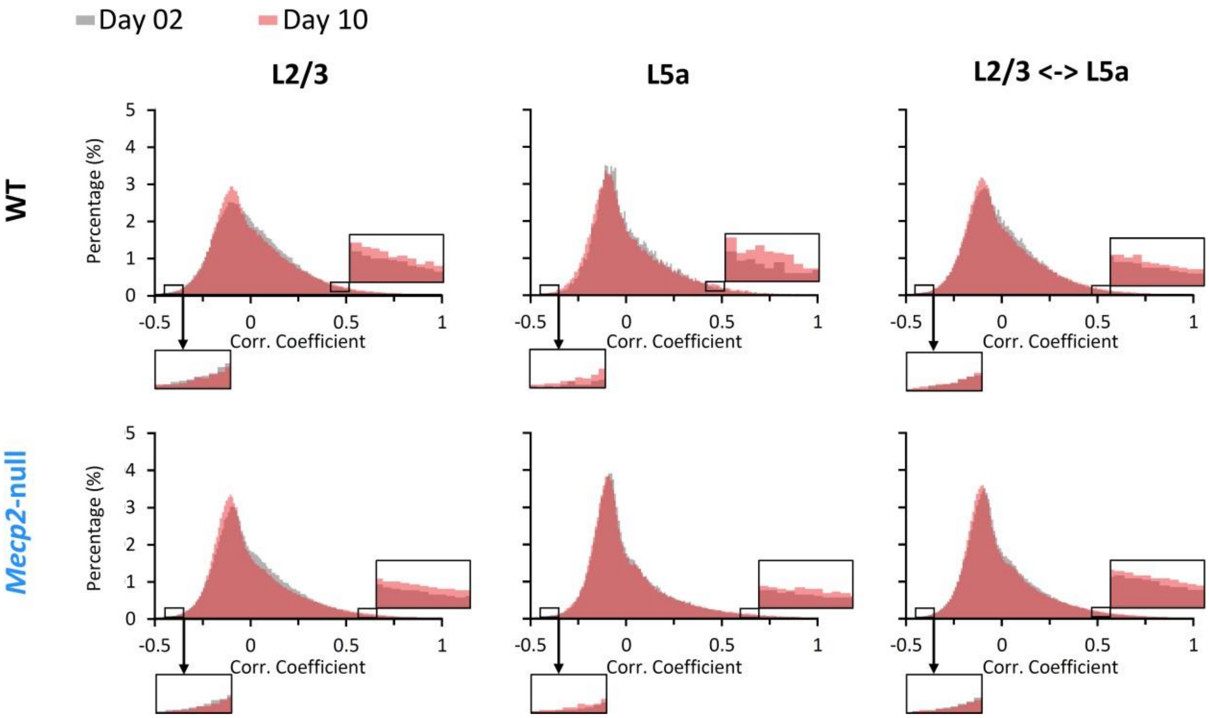

Fig. S8. The distributions of Pearson's correlation coefficients of functional neuronal pairs are very wide, reflecting the dynamism of M1. L2/3 WT, n=11 mice;L2/3 *Mecp2*-null, n=12 mice; L5a WT, n=8 mice; L5a *Mecp2*-null, n=10 mice (we excluded L5a data from mice from whom we detected fewer than 20 neurons in L5a). Across both layers, WT, n=8 mice; *Mecp2*-null, n=10 mice.

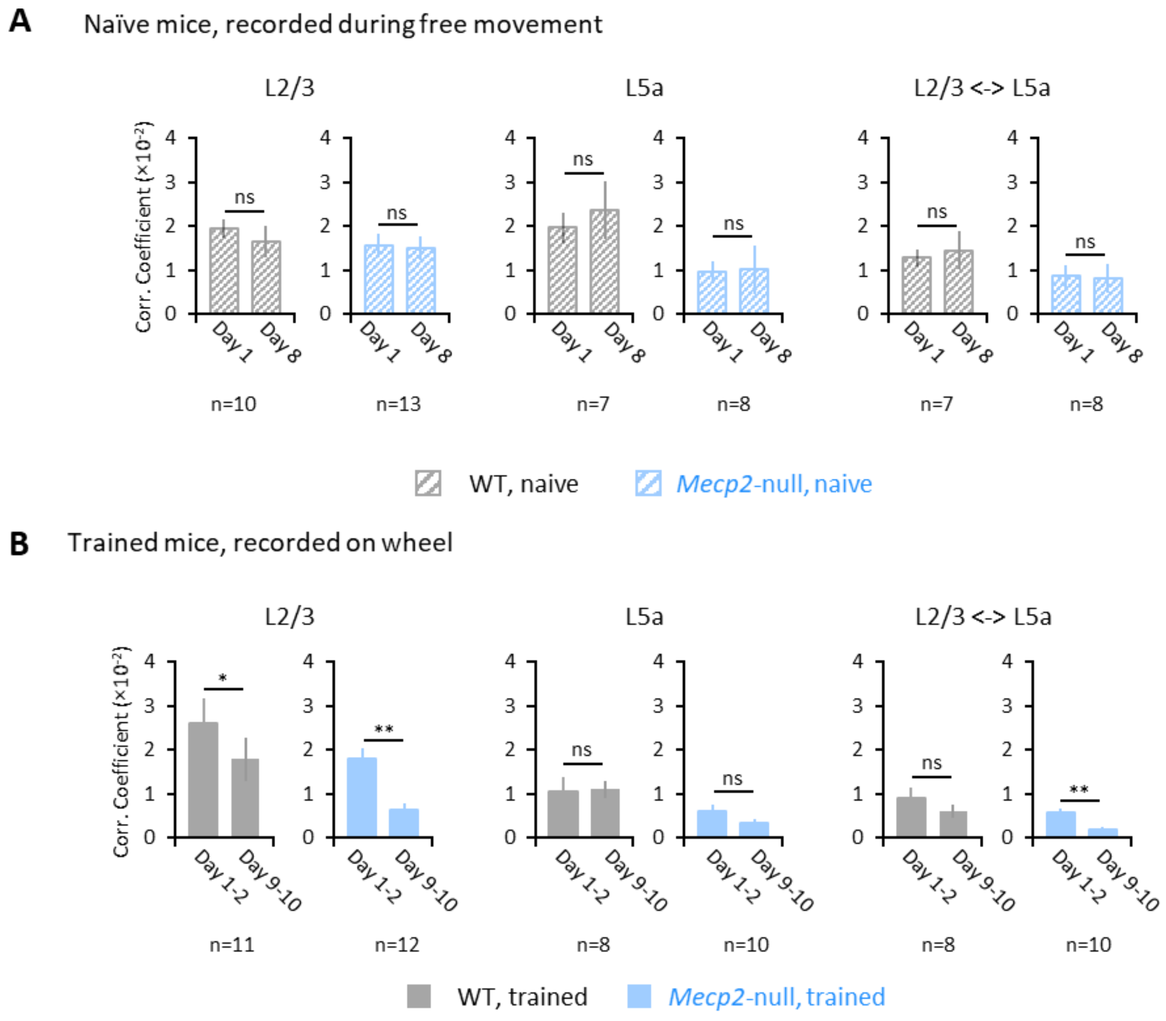

Fig. S9. Circuit dynamics in naïve mice during free-running did not change over the course of a week, but the circuit in trained mice did. (**A**) Comparison of averaged correlation coefficients during free-run mode between days 1 and 8 in naive WT and *Mecp2*-null mice. There is no difference between days. (**B**) Comparison of averaged correlation coefficients during free-run mode between days 1-2 and 9-10 in trained WT and *Mecp2*-null mice. This indicates that the changes we observe in the M1 circuit over the course of learning do not just correlate with the behavioral improvements, but have a causal relationship to those improvements. Error bars represent mean ± SE. ns, not significant; * $P < 0.05$, ** $P < 0.01$, Wilcoxon signed-rank test.

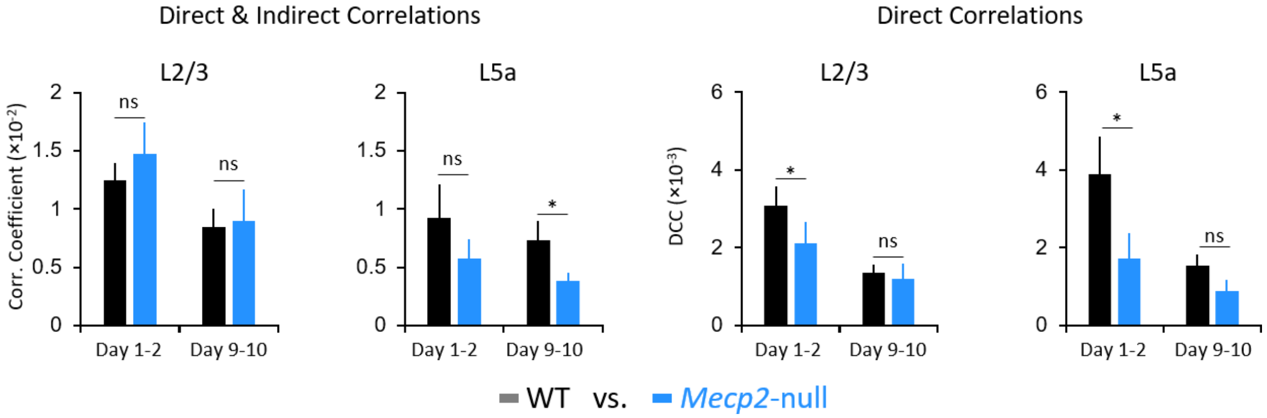

Fig. S10. Cross-group comparison of Pearson's correlation coefficient. Comparison of averaged correlation coefficients between WT and *Mecp2*-null mice during days 1-2 (learning) and days 9-10 (consolidation). The same data set was used as in **Fig 3**. L2/3 WT, n=11 mice; L2/3 Mecp2-null, n=12mice; L5a WT, n=8 mice; L5a Mecp2-null, n=10 mice. Error bars represent mean ± SE. ns, not significant; * $P < 0.05$, Mann-Whitney U Test.

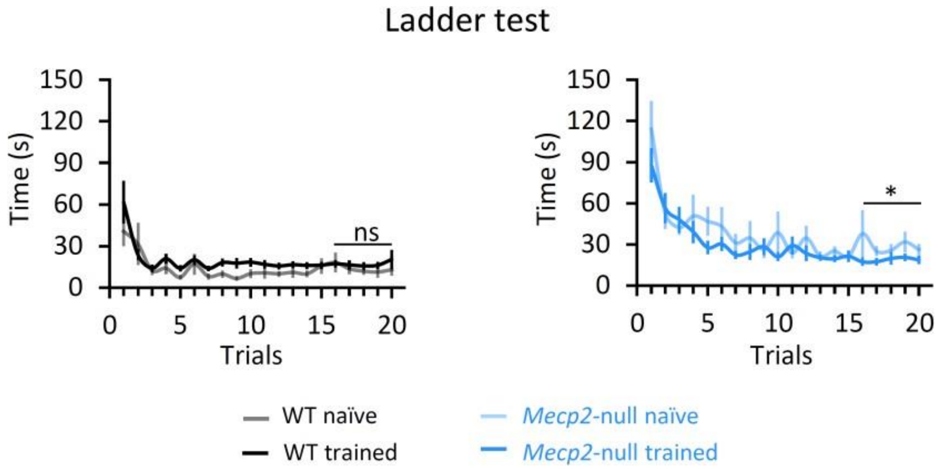

Fig. S11. Wheel-speed-training improved the performance of *Mecp2* null mice on the ladder test. Graphs show the duration of time wild-type and null mice needed to cross the ladder from one end to the other over the course of 20 trials. The less time spent on the ladder, the better the mouse's performance. Naive: WT: n=15 mice; *Mecp2*-null, n=17 mice; Trained: WT: n=19 mice; *Mecp2*-null, n=17 mice. Error bars represent mean ± SE. ns, not significant, * $P< 0.05$, RM-ANOVA test.

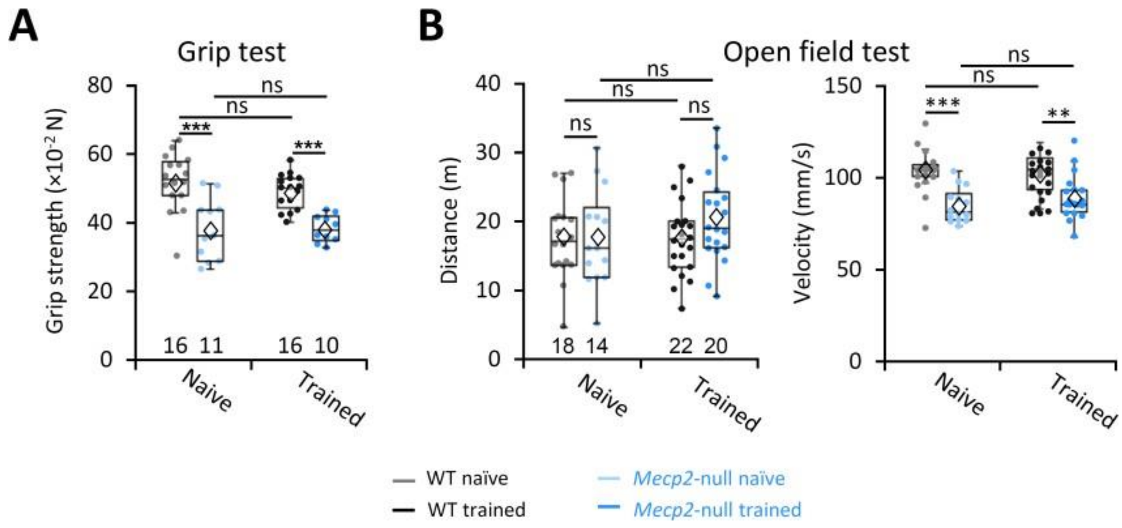

Fig. S12. Motor training did not improve strength on the grip test or change parameters of physical performance on the open field test. (**A**) Summary of the grip strength of front limbs. Naive: WT: n=16 mice; *Mecp2*-null, n=11 mice; Trained: WT: n=16 mice; *Mecp2*-null, n=10 mice. Error bars represent mean ± SE. ns, not significant, * $P < 0.05$, ** $P < 0.01$, *** $P < 0.001$, Two-way ANOVA test with Sidak's Post-Hoc. Diamond symbol represents the mean value. (**B**) Distance traveled in the open field testwas not affected by genotype; the null mice were slower than WT in moving around, regardless of training. The number of animals in each group is indicated beneath each bar. Naive: WT: n=18 mice; *Mecp2*-null, n=14 mice; Trained: WT: n=22 mice; *Mecp2*-null, n=20mice. Error bars represent mean ± SE. ns, not significant, ** $P < 0.01$, *** $P < 0.001$, Two-way ANOVA test with Sidak's Post-Hoc.

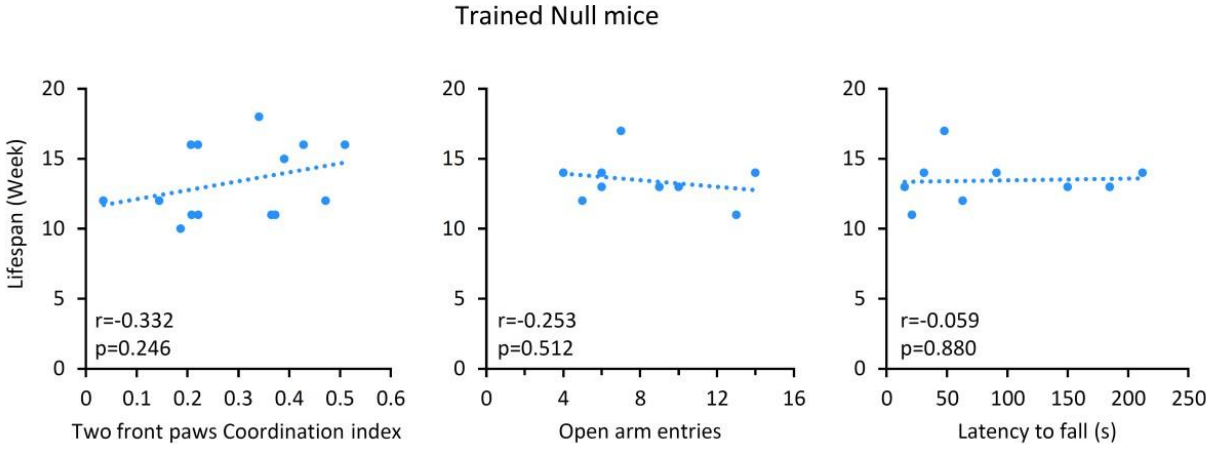

Fig. S13. Longer lifespan correlates with motor learning, but not with reduced anxiety-like behavior or stamina on the rotarod. Although the correlation between the lifespan and improved forepaw coordination is weak (p = 0.236 and r = 0.336, Pearson bivariate correlations test), it suggests that motor learning contributes to extending the survival of *Mecp2*-null mice.

Supplemental table 1: Detailed statistical results for each experiment, organized by figure panel.

	Figure. Panel	Test type	Sample size (mouse)	Group-wise comparison	Test stat.	DF	P-value	Asterisk
Stride number	Fig. 1D	RM-ANOVA test; Sidak's Post-Hoc	WT: 15; Null: 16	WT vs. Null	F = 59.8	dfn=1, dfd=29	0.000	***
				Speed 15: WT vs. Null			0.000	***
				Speed 30: WT vs. Null			0.000	***
				Speed 45: WT vs. Null			0.000	***
				Speed 60: WT vs. Null			0.000	***
Stride number	Fig. 1E left panel	RM-ANOVA test	WT: 15; Null: 16	WT vs. Null	F = 69.4	dfn=1, dfd=29	0.000	***
	Fig. 1E rigtht panel	Wilcoxon signed-rank test	WT: 15	Day 2 vs. Day 10	Z = -3.124		0.002	**
		Wilcoxon signed-rank test	Null: 16	Day 2 vs. Day 10	Z = -1.656		0.098	ns
Stride length	Fig. 1F left panel	RM-ANOVA test	WT: 15; Null: 16	WT vs. Null	F =0.008	dfn=1, dfd=29	0.928	ns
	Fig. 1F rigtht panel	Wilcoxon signed-rank test	WT: 15	Day 2 vs. Day 10	Z = 2.215		0.027	*
		Wilcoxon signed-rank test	Null: 16	Day 2 vs. Day 10	F = 2.372		0.020	*
Paw coordination	Fig. 1H left panel	RM-ANOVA test	WT: 15; Null: 16	Coordination index: WT vs. Null	F = 6.546	dfn=1, dfd=29	0.016	*
	Fig. 1H right panel	Wilcoxon signed-rank test	WT: 15	Day 2 vs. Day 10	Z = 2.556		0.011	*
		Wilcoxon signed-rank test	Null: 16	Day 2 vs. Day 10	F = 3.361		0.001	***
Event rate of each speeds	Fig. 2C	RM-ANOVA test; Sidak's Post-Hoc	WT: 13; Null: 14	L2/3: WT vs. Null	F = 6.4	dfn=1, dfd=25	0.018	*
				L2/3 Speed 0: WT vs. Null			0.281	ns
				L2/3 Speed 15: WT vs. Null			0.005	**
				L2/3 Speed 30: WT vs. Null			0.001	**

				L2/3 Speed 45: WT vs. Null			0.004	**
				L2/3 Speed 60: WT vs. Null			0.001	**
			WT: 11; Null: 13	L5a: WT vs. Null	F = 3.7	dfn=1, dfd=22	0.065	ns
				L5a Speed 0: WT vs. Null			0.656	ns
				L5a Speed 15: WT vs. Null			0.069	ns
				L5a Speed 30: WT vs. Null			0.045	*
				L5a Speed 45: WT vs. Null			0.019	*
				L5a Speed 60: WT vs. Null			0.016	*
Event rate of each day	Fig. 2D	RM-ANOVA test	WT: 13; Null: 14	L2/3 Speed 0: WT vs. Null	F = 0.008	dfn=1, dfd=25	0.931	ns
		RM-ANOVA test	WT: 13; Null: 14	L2/3 Speed 30: WT vs. Null	F = 9.168	dfn=1, dfd=25	0.006	**
		RM-ANOVA test	WT: 13; Null: 14	L2/3 Speed 60: WT vs. Null	F = 9.521	dfn=1, dfd=25	0.005	**
		RM-ANOVA test	WT: 11; Null: 13	L5a Speed 0: WT vs. Null	F = 1.056	dfn=1, dfd=22	0.315	ns
		RM-ANOVA test	WT: 11; Null: 13	L5a Speed 30: WT vs. Null	F = 4.301	dfn=1, dfd=22	0.050	*
		RM-ANOVA test	WT: 11; Null: 13	L5a Speed 60: WT vs. Null	F = 5.350	dfn=1, dfd=22	0.030	*
Response to speed changes	Fig. 2E	Kolmogorov–Smirnov test	11	L2/3 WT Speed 0->15: Day 2 vs. Day 10			0.000	*
		Kolmogorov–Smirnov test	11	L2/3 WT Speed 45->60: Day 2 vs. Day 10			0.015	ns
		Kolmogorov–Smirnov test	12	L2/3 Null Speed 0->15: Day 2 vs. Day 10			0.000	*
		Kolmogorov–Smirnov test	12	L2/3 Null Speed 45->60:			0.000	*

				Day 2 vs. Day 10				
		Kolmogorov–Smirnov test	8	L5a WT Speed 0->15: Day 2 vs. Day 10			0.000	*
		Kolmogorov–Smirnov test	8	L5a WT Speed 45->60: Day 2 vs. Day 10			0.000	*
		Kolmogorov–Smirnov test	10	L5a Null Speed 0->15: Day 2 vs. Day 10			0.148	ns
		Kolmogorov–Smirnov test	10	L5a Null Speed 45->60: Day 2 vs. Day 10			0.000	*
Event rate during 10s after transition to Speed 60	Fig. 2F	RM-ANOVA test	WT: 11; Null: 12;	L2/3: WT vs. Null	F = 8.946	dfn=1, dfd=21	0.007	**
		RM-ANOVA test	WT: 8; Null: 10	L5a: WT vs. Null	F = 1.223	dfn=1, dfd=16	0.284	ns
Direct & Indirect Correlations	Fig. 3A	Wilcoxon signed-rank test	11	All pairs L2/3 WT: Day 1-2 vs. Day 9-10	Z = -2.3		0.021	*
		Wilcoxon signed-rank test	12	All pairs L2/3 Null: Day 1-2 vs. Day 9-10	Z = -2.3		0.023	*
		Wilcoxon signed-rank test	8	All pairs L5a WT: Day 1-2 vs. Day 9-10	Z = -0.8		0.401	ns
		Wilcoxon signed-rank test	10	All pairs L5a Null: Day 1-2 vs. Day 9-10	Z = -1.2		0.241	ns
		Wilcoxon signed-rank test	8	All pairs L2/3<->L5a WT: Day 1-2 vs. Day 9-10	Z = -2.2		0.025	*
		Wilcoxon signed-rank test	10	All pairs L2/3<->L5a Null: Day 1-2 vs. Day 9-10	Z = -2.7		0.007	**
		Wilcoxon signed-rank test	11	Strongly correlated pairs L2/3 WT: Day 1-	Z = -2.7		0.008	**

				2 vs. Day 9-10				
		Wilcoxon signed-rank test	12	Strongly correlated pairs L2/3 Null: Day 1-2 vs. Day 9-10	Z = -2.8		0.005	**
		Wilcoxon signed-rank test	8	Strongly correlated pairs L5a WT: Day 1-2 vs. Day 9-10	Z = -2.1		0.036	*
		Wilcoxon signed-rank test	10	Strongly correlated pairs L5a Null: Day 1-2 vs. Day 9-10	Z = -2.8		0.005	**
		Wilcoxon signed-rank test	8	Strongly correlated pairs L2/3<->L5a WT: Day 1-2 vs. Day 9-10	Z = -2.5		0.012	*
		Wilcoxon signed-rank test	10	Strongly correlated pairs L2/3<->L5a Null: Day 1-2 vs. Day 9-10	Z = -2.8		0.005	**
Direct Correlations	Fig. 3B	Wilcoxon signed-rank test	11	All pairs L2/3 WT: Day 1-2 vs. Day 9-10	Z = -2.8		0.006	**
		Wilcoxon signed-rank test	12	All pairs L2/3 Null: Day 1-2 vs. Day 9-10	Z = -3.1		0.002	**
		Wilcoxon signed-rank test	8	All pairs L5a WT: Day 1-2 vs. Day 9-10	Z = -2.2		0.025	*
		Wilcoxon signed-rank test	10	All pairs L5a Null: Day 1-2 vs. Day 9-10	Z = -1.4		0.169	ns
		Wilcoxon signed-rank test	8	All pairs L2/3<->L5a WT: Day 1-2 vs. Day 9-10	Z = -2.5		0.012	*

		Wilcoxon signed-rank test	10	All pairs L2/3<->L5a Null: Day 1-2 vs. Day 9-10	Z = -2.7		0.007	**
		Wilcoxon signed-rank test	11	Strongly correlated pairs L2/3 WT: Day 1-2 vs. Day 9-10	Z = -2.8		0.004	**
		Wilcoxon signed-rank test	12	Strongly correlated pairs L2/3 Null: Day 1-2 vs. Day 9-10	Z = -2.4		0.019	*
		Wilcoxon signed-rank test	8	Strongly correlated pairs L5a WT: Day 1-2 vs. Day 9-10	Z = -2.4		0.017	*
		Wilcoxon signed-rank test	10	Strongly correlated pairs L5a Null: Day 1-2 vs. Day 9-10	Z = -0.6		0.508	ns
		Wilcoxon signed-rank test	8	Strongly correlated pairs L2/3<->L5a WT: Day 1-2 vs. Day 9-10	Z = -2.2		0.025	*
		Wilcoxon signed-rank test	10	Strongly correlated pairs L2/3<->L5a Null: Day 1-2 vs. Day 9-10	Z = -2.3		0.022	*
Cross-layer Correlations	Fig. 3C	RM-ANOVA test	WT: 8; Null: 10	WT vs. Null	F = 6.932	dfn=1, dfd=16	0.018	*
Fraction of directly-correlated pairs (%)	Fig. 4C	Student's t-test	WT: 9; Null: 9	Rest: WT vs. Null	F = 0.758	16	0.035	*
				Run: WT vs. Null	F = 3.067	16	0.031	*
DCC100 / DCC200	Fig. 4F	RM-ANOVA test	WT: 11; Null: 12	L2/3: WT vs. Null	F = 7.133	dfn=1, dfd=21	0.014	*
		RM-ANOVA test	WT: 8; Null: 10	L5a: WT vs. Null	F = 6.911	dfn=1, dfd=16	0.018	*

Duration on ladder	Fig. 5B	RM-ANOVA test	WT: 15; Null: 17	Naive: WT vs. Null	F = 12.880	dfn=1, dfd=30	0.001	***
		RM-ANOVA test	WT: 19; Null: 17	Trained: WT vs. Null	F = 0.579	dfn=1, dfd=34	0.452	ns
Short steps	Fig. 5C	RM-ANOVA test	Naïve: 17; Training: 17;	Short Step: Naïve vs. Trained	F = 9.463	dfn=1, dfd=32	0.004	**
Irregular steps		RM-ANOVA test	Naïve: 17; Training: 17;	Irregular Step: Naïve vs. Trained	F = 21.901	dfn=1, dfd=32	0.000	***
Duration on ladder	Fig. 5D	One-way ANOVA test; Fisher's LSD post hoc	Naïve: 17; 2 day after training: 11; 4 day after training: 6	Between Groups	F = 3.4	33	0.048	*
				Duration Naïve vs. 2 day after training			0.041	*
				Duration Naïve vs. 4 day after training			0.045	*
				Duration 2 day after training vs. 4 day after training			0.744	ns
Short steps		One-way ANOVA test; Fisher's LSD post hoc	Naïve: 17; 2 day after training: 11; 4 day after training: 6	Between Groups	F = 4.2	33	0.025	*
				Short Step Naïve vs. 2 day after training			0.019	*
				Short Step Naïve vs. 4 day after training			0.034	*
				Short Step 2 day after training vs. 4 day after training			0.854	ns
Irregular steps		One-way ANOVA test; Fisher's LSD post hoc	Naïve: 17; 2 day after training: 11; 4 day after training: 6	Between Groups	F = 15.18	33	0.000	***
				Irregular Step Naïve vs. 2 day after training			0.000	***
				Irregular Naïve vs. 4 day after training			0.001	***
				Irregular 2 day after training vs. 4			0.583	ns

				day after training				
Latency to fall, trial 1 (in seconds)	Fig. 5E	Two-way ANOVA test; Sidak's Post-Hoc	WT Naïve: 13; WT Trained: 13; Null Naïve: 9; Null Trained: 10	WT vs. Null	F = 92.477	dfn=1, dfd=41	0.000	***
				Naïve: WT vs. Null			0.000	***
				Trained: WT vs. Null			0.000	***
				Naive vs. Trained	F = 8.387	dfn=1, dfd=41	0.006	**
				WT: Naive vs. Trained			0.041	*
				Null: Naive vs. Trained			0.050	*
Latency to stop locomoting (in seconds)	Fig. 5F	RM-ANOVA test	WT Naïve: 11; WT Training: 9;	WT: Naive vs. Trained	F =5.335	dfn=1, dfd=18	0.033	*
			Null Naïve: 7; Null Training: 8	Null: Naive vs. Trained	F = 8.212	dfn=1, dfd=13	0.013	*
			WT Naïve: 11; Null Naïve: 7	Naive: WT vs. Null	F = 72.906	dfn=1, dfd=16	0.000	***
			WT Training: 9; Null Training: 8	Trained: WT vs. Null	F = 88.963	dfn=1, dfd=15	0.000	***
Fold-change of latency to stop locomoting		RM-ANOVA test	Fold of change: WT: 9; Null: 8	WT vs. Null	F = 9.369	dfn=1, dfd=15	0.008	**
Open field test duration in center (%)	Fig. 6A	Two-way ANOVA test; Sidak's Post-Hoc	WT Naïve: 18; WT Trained: 22; Null Naïve: 14; Null Trained: 20	Duration in center: WT vs. Null	F = 1.693	dfn=1, dfd=70	0.197	ns
				Duration in center: Naïve; WT vs. Null			0.043	*
				Duration in center: Trained; WT vs. Null			0.696	ns
				Duration in center: Naive vs. Trained	F = 5.008	dfn=1, dfd=70	0.028	*
				Duration in center: WT; Naïve vs. Trained			0.755	ns
				Duration in center: Null;			0.008	**

				Naïve vs. Trained				
Open field test center entries		Two-way ANOVA test; Sidak's Post-Hoc	WT Naïve: 18; WT Trained: 22; Null Naïve: 14; Null Trained: 20	Center entries: WT vs. Null	F = 6.268	dfn=1, dfd=70	0.015	*
				Center entries: Naïve; WT vs. Null			0.004	**
				Center entries: Trained; WT vs. Null			0.694	ns
				Center entries: Naive vs. Trained	F = 4.006	dfn=1, dfd=70	0.049	*
				Center entries: WT; Naïve vs. Trained			0.991	ns
				Center entries: Null; Naïve vs. Trained			0.009	**
Elevated plus maze test duration in open arms (%)	Fig. 6B	Two-way ANOVA test; Sidak's Post-Hoc	WT Naïve: 12; WT Trained: 11; Null Naïve: 8; Null Trained: 16	Duration in open arms: WT vs. Null	F = 30.16	dfn=1, dfd=43	0.000	***
				Duration in open arms: Naïve: WT vs. Null			0.015	*
				Duration in open arms: Trained: WT vs. Null			0.000	***
				Duration in open arms: Naïve vs. Trained	F = 2.403	dfn=1, dfd=43	0.128	ns
				Duration in open arms: WT: Naïve vs. Trained			0.951	ns
				Duration in open arms: Null: Naïve vs. Trained			0.032	*
Elevated plus maze test, ratio of entries in		Two-way ANOVA; Sidak's Post-Hoc	WT Naïve: 12; WT Trained: 11; Null Naïve: 8;	Entries ratio: WT vs. Null	F = 5.700	dfn=1, dfd=43	0.021	*
				Entries ratio: Naïve: WT vs. Null			0.516	ns

open over closed arms			Null Trained: 16	Entries ratio: Trained: WT vs. Null			0.006	**
				Entries ratio: Naïve vs. Trained	F = 5.157	dfn=1, dfd=43	0.028	*
				Entries ratio: WT: Naïve vs. Trained			0.531	ns
				Entries ratio: Null: Naïve vs. Trained			0.015	*
Survival of Mecp2-null mice	Fig. 6C	Mann-Whitney U test	Naïve: 25; Trained: 20	Naïve vs. Trained	U = 382		0.002	**
degree of improvement in stride and coordination between week 1 and week 2	Fig. S1C	Wilcoxon signed-rank test	15	Stride number WT: Day 2 vs. Day 7	Z = -2.3		0.020	*
		Wilcoxon signed-rank test	15	Stride number Null: Day 2 vs. Day 7	Z = -3.2		0.001	**
		Wilcoxon signed-rank test	15	Stride number WT: Day 8 vs. Day 12	Z = -1.2		0.222	ns
		Wilcoxon signed-rank test	12	Stride number Null: Day 8 vs. Day 12	Z = 1.0		0.308	ns
		Wilcoxon signed-rank test	15	Stride length WT: Day 2 vs. Day 7	Z = 1.5		0.125	ns
		Wilcoxon signed-rank test	15	Stride length Null: Day 2 vs. Day 7	Z = 2.0		0.044	*
		Wilcoxon signed-rank test	15	Stride length WT: Day 8 vs. Day 12	Z = 0.1		0.865	ns
		Wilcoxon signed-rank test	12	Stride length Null: Day 8 vs. Day 12	Z = 1.1		0.272	ns
		Wilcoxon signed-rank test	15	Coordination index WT: Day 2 vs. Day 7	Z = 1.9		0.053	ns
		Wilcoxon signed-rank test	16	Coordination index Null: Day 2 vs. Day 7	Z = 3.5		0.000	***
		Wilcoxon signed-rank test	15	Coordination index WT: Day 8 vs. Day 12	Z = -1.1		0.256	ns

		Wilcoxon signed-rank test	15	Coordination index Null: Day 8 vs. Day 12	Z = 1.8		0.078	ns
Soma size	Fig. S2B	Rank sum test	L2/3: 28125 neurons; L5a: 10395 neurons	L2/3 vs. L5a			0.000	***
Event rate	Fig. S3	RM-ANOVA test	WT: 13; Null: 14	Increasing speed: Speed 0: WT vs. Null	F = 0.008	dfn=1, dfd=25	0.931	ns
		RM-ANOVA test	WT: 13; Null: 14	Increasing speed: Speed 15: WT vs. Null	F = 5.179	dfn=1, dfd=25	0.032	*
		RM-ANOVA test	WT: 13; Null: 14	Increasing speed: Speed 30: WT vs. Null	F = 9.168	dfn=1, dfd=25	0.006	**
		RM-ANOVA test	WT: 13; Null: 14	Increasing speed: Speed 45: WT vs. Null	F = 6.164	dfn=1, dfd=25	0.020	*
		RM-ANOVA test	WT: 13; Null: 14	Increasing speed: Speed 60: WT vs. Null	F = 9.521	dfn=1, dfd=25	0.005	**
		RM-ANOVA test	WT: 13; Null: 14	Decreasing speed: Speed 0: WT vs. Null	F = 3.463	dfn=1, dfd=25	0.075	ns
		RM-ANOVA test	WT: 13; Null: 14	Decreasing speed: Speed 15: WT vs. Null	F = 7.326	dfn=1, dfd=25	0.012	*
		RM-ANOVA test	WT: 13; Null: 14	Decreasing speed: Speed 30: WT vs. Null	F = 12.312	dfn=1, dfd=25	0.002	**
		RM-ANOVA test	WT: 13; Null: 14	Decreasing speed: Speed 45: WT vs. Null	F = 12.936	dfn=1, dfd=25	0.001	**
		RM-ANOVA test	WT: 13; Null: 14	Decreasing speed: Speed 60: WT vs. Null	F =11.211	dfn=1, dfd=25	0.003	**
Event rate	Fig. S4	RM-ANOVA test	WT: 11; Null: 13	Increasing speed: Speed 0: WT vs. Null	F = 1.056	dfn=1, dfd=22	0.315	ns
		RM-ANOVA test	WT: 11; Null: 13	Increasing speed: Speed	F = 2.110	dfn=1, dfd=22	0.160	ns

				15: WT vs. Null				
		RM-ANOVA test	WT: 11; Null: 13	Increasing speed: Speed 30: WT vs. Null	F = 4.301	dfn=1, dfd=22	0.050	*
		RM-ANOVA test	WT: 11; Null: 13	Increasing speed: Speed 45: WT vs. Null	F = 4.817	dfn=1, dfd=22	0.039	*
		RM-ANOVA test	WT: 11; Null: 13	Increasing speed: Speed 60: WT vs. Null	F = 5.350	dfn=1, dfd=22	0.030	*
		RM-ANOVA test	WT: 11; Null: 13	Decreasing speed: Speed 0: WT vs. Null	F = 1.160	dfn=1, dfd=22	0.293	ns
		RM-ANOVA test	WT: 11; Null: 13	Decreasing speed: Speed 15: WT vs. Null	F = 6.064	dfn=1, dfd=22	0.022	*
		RM-ANOVA test	WT: 11; Null: 13	Decreasing speed: Speed 30: WT vs. Null	F = 3.803	dfn=1, dfd=22	0.064	ns
		RM-ANOVA test	WT: 11; Null: 13	Decreasing speed: Speed 45: WT vs. Null	F = 8.308	dfn=1, dfd=22	0.009	**
		RM-ANOVA test	WT: 11; Null: 13	Decreasing speed: Speed 60: WT vs. Null	F = 9.269	dfn=1, dfd=22	0.006	**
Event rate	Fig. S5A	Wilcoxon signed-rank test	11	Rest L2/3 WT: Day 1-2 vs. Day 9-10	Z = -1.334		0.182	ns
		Wilcoxon signed-rank test	12	Rest L2/3 Null: Day 1-2 vs. Day 9-10	Z = -1.334		0.182	ns
		Wilcoxon signed-rank test	8	Rest L5a WT: Day 1-2 vs. Day 9-10	Z = -0.700		0.484	ns
		Wilcoxon signed-rank test	10	Rest L5a Null: Day 1-2 vs. Day 9-10	Z = -1.274		0.203	ns
		Wilcoxon signed-rank test	11	Run L2/3 WT: Day 1-2 vs. Day 9-10	Z = -2.578		0.010	*

		Wilcoxon signed-rank test	12	Run L2/3 Null: Day 1-2 vs. Day 9-10	Z = -2.275		0.023	*
		Wilcoxon signed-rank test	8	Run L5a WT: Day 1-2 vs. Day 9-10	Z = -0.280		0.779	ns
		Wilcoxon signed-rank test	10	Run L5a Null: Day 1-2 vs. Day 9-10	Z = -1.172		0.241	ns
Event rate distribution	Fig. S5B	Kolmogorov–Smirnov test	13	L2/3 WT Speed 0: Day 2 vs. Day 10			0.000	*
		Kolmogorov–Smirnov test	13	L2/3 WT Speed 60: Day 2 vs. Day 10			0.000	*
		Kolmogorov–Smirnov test	14	L2/3 Null Speed 0: Day 2 vs. Day 10			0.000	*
		Kolmogorov–Smirnov test	14	L2/3 Null Speed 60: Day 2 vs. Day 10			0.000	*
		Kolmogorov–Smirnov test	11	L5a WT Speed 0: Day 2 vs. Day 10			0.001	ns
		Kolmogorov–Smirnov test	11	L5a WT Speed 60: Day 2 vs. Day 10			0.001	ns
		Kolmogorov–Smirnov test	13	L5a Null Speed 0: Day 2 vs. Day 10			0.000	*
		Kolmogorov–Smirnov test	13	L5a Null Speed 60: Day 2 vs. Day 10			0.000	*
ER width	Fig. S5C	RM-ANOVA test	13	L2/3 WT: Speed 0 vs. Speed 60	F = 0.813	dfn=1, dfd=24	0.376	ns
		RM-ANOVA test	14	L2/3 Null: Speed 0 vs. Speed 60	F = 6.121	dfn=1, dfd=26	0.020	*
		RM-ANOVA test	11	L5a WT: Speed 0 vs. Speed 60	F = 0.473	dfn=1, dfd=20	0.499	ns

		RM-ANOVA test	13	L5a Null: Speed 0 vs. Speed 60	F = 0.191	dfn=1, dfd=24	0.666	ns
Probability of response change	Fig. S6D	Two-tailed t-test	WT: 7; Null: 5	Probability of response change: WT vs. Null	t = -2.054	10	0.069	ns
Rest Corr. Coefficient (×10-2)	Fig. S7A	Wilcoxon signed-rank test	11	All pairs L2/3 WT: Day 1-2 vs. Day 9-10	Z = -0.889		0.374	ns
		Wilcoxon signed-rank test	12	All pairs L2/3 Null: Day 1-2 vs. Day 9-10	Z = -2.510		0.012	*
		Wilcoxon signed-rank test	8	All pairs L5a WT: Day 1-2 vs. Day 9-10	Z = -0.280		0.779	ns
		Wilcoxon signed-rank test	10	All pairs L5a Null: Day 1-2 vs. Day 9-10	Z = 0.968		0.333	ns
		Wilcoxon signed-rank test	8	All pairs L2/3<->L5a WT: Day 1-2 vs. Day 9-10	Z = -0.840		0.401	ns
		Wilcoxon signed-rank test	10	All pairs L2/3<->L5a Null: Day 1-2 vs. Day 9-10	Z = -0.459		0.646	ns
		Wilcoxon signed-rank test	11	Strongly correlated pairs L2/3 WT: Day 1-2 vs. Day 9-10	Z = 0.889		0.374	ns
		Wilcoxon signed-rank test	12	Strongly correlated pairs L2/3 Null: Day 1-2 vs. Day 9-10	Z = -1.883		0.060	ns
		Wilcoxon signed-rank test	8	Strongly correlated pairs L5a WT: Day 1-2 vs. Day 9-10	Z = -0.980		0.327	ns
		Wilcoxon signed-rank test	10	Strongly correlated pairs L5a Null: Day 1-	Z = 0.764		0.445	ns

				2 vs. Day 9-10				
		Wilcoxon signed-rank test	8	Strongly correlated pairs L2/3<->L5a WT: Day 1-2 vs. Day 9-10	Z = -0.420		0.674	ns
		Wilcoxon signed-rank test	10	Strongly correlated pairs L2/3<->L5a Null: Day 1-2 vs. Day 9-10	Z = -0.255		0.799	ns
All pairs Corr. Coefficient of navie mice during free run	Fig. S9A	Wilcoxon signed-rank test	10	L2/3 WT: Day 1 vs. Day 8	Z = -0.866		0.386	ns
		Wilcoxon signed-rank test	13	L2/3 Null: Day 1 vs. Day 8	Z = -0.245		0.807	ns
		Wilcoxon signed-rank test	7	L5a WT: Day 1 vs. Day 8	Z = -1.014		0.310	ns
		Wilcoxon signed-rank test	8	L5a Null: Day 1 vs. Day 8	Z = -0.840		0.401	ns
		Wilcoxon signed-rank test	7	L2/3<->L5a WT: Day 1 vs. Day 8	Z = -0.845		0.398	ns
		Wilcoxon signed-rank test	8	L2/3<->L5a Null: Day 1 vs. Day 8	Z = -0.140		0.889	ns
All pairs Corr. Coefficient of Trained mice during free run	Fig. S9B	Wilcoxon signed-rank test	11	L2/3 WT: Day 1-2 vs. Day 9-10	Z = -2.134		0.033	*
		Wilcoxon signed-rank test	12	L2/3 Null: Day 1-2 vs. Day 9-10	Z = -3.059		0.002	**
		Wilcoxon signed-rank test	8	L5a WT: Day 1-2 vs. Day 9-10	Z = -0.280		0.779	ns
		Wilcoxon signed-rank test	10	L5a Null: Day 1-2 vs. Day 9-10	Z = -1.784		0.074	ns
		Wilcoxon signed-rank test	8	L2/3<->L5a WT: Day 1-2 vs. Day 9-10	Z = -1.820		0.069	ns
Direct & Indirect Correlations	Fig. S10	Mann-Whitney U Test	WT Naive: 11; Null Naive: 12	L2/3: WT Naïve vs. Null Naïve	U = 71.000		0.786	ns

		Mann-Whitney U Test	WT Trained: 11; Null Trained: 12	L2/3: WT Trained vs. Null Trained	U = 57.000		0.608	ns
		Mann-Whitney U Test	WT Naive: 8; Null Naive: 10	L5a: WT Naïve vs. Null Naïve	U = 24.000		0.786	ns
		Mann-Whitney U Test	WT Trained: 8; Null Trained: 10	L5a: WT Trained vs. Null Trained	U = 16.000		0.034	*
Direct Correlations		Mann-Whitney U Test	WT Naive: 11; Null Naive: 12	L2/3: WT Naïve vs. Null Naïve	U = 33.000		0.044	*
		Mann-Whitney U Test	WT Trained: 11; Null Trained: 12	L2/3: WT Trained vs. Null Trained	U = 42.000		0.151	ns
		Mann-Whitney U Test	WT Naive: 8; Null Naive: 10	L5a: WT Naïve vs. Null Naïve	U = 17.000		0.043	*
		Mann-Whitney U Test	WT Trained: 8; Null Trained: 10	L5a: WT Trained vs. Null Trained	U = 20.000		0.083	ns
Ladder test	Fig. S11	RM-ANOVA test	WT Naïve: 15; WT Training: 19;	WT: Naive vs. Trained	F = 2.018	dfn=1, dfd=32	0.165	ns
		RM-ANOVA test	Null Naïve: 17; Null Training: 17	Null: Naive vs. Trained	F = 6.710	dfn=1, dfd=32	0.014	*
Grip test	Fig. S12A	Two-way ANOVA; Sidak's Post-Hoc	WT Naïve: 16; WT Trained: 16; Null Naïve: 11; Null Trained: 10	WT vs. Null	F = 39.841	dfn=1, dfd=49	0.000	***
				Naïve: WT vs. Null			0.000	***
				Trained: WT vs. Null			0.000	***
				Naïve vs. Trained	F = 0.478	dfn=1, dfd=49	0.493	ns
				WT: Naïve vs. Trained			0.254	ns
				Null: Naïve vs. Trained			0.964	ns
Open field test Distance (m)	Fig. S12B	Two-way ANOVA; Sidak's Post-Hoc	WT Naïve: 18; WT Trained: 22; Null Naïve: 14; Null Trained: 20	Distance: WT vs. Null	F = 1.187	dfn=1, dfd=70	0.280	ns
				Distance: Naïve: WT vs. Null			0.955	ns
				Distance: Trained: WT vs. Null			0.114	ns

				Distance: Naive vs. Trained	F = 0.975	dfn=1, dfd=70	0.327	ns
				Distance: WT: Naive vs. Trained			0.990	ns
				Distance: Null: Naive vs. Trained			0.182	ns
Open field test Velocity (mm/s)		Two-way ANOVA; Sidak's Post-Hoc	WT Naïve: 18; WT Trained: 22; Null Naïve: 14; Null Trained: 20	Velocity: WT vs. Null	F = 34.374	dfn=1, dfd=70	0.000	***
				Velocity: Naïve: WT vs. Null			0.000	***
				Velocity: Trained: WT vs. Null			0.002	**
				Velocity: Naive vs. Trained	F = 0.006	dfn=1, dfd=70	0.940	ns
				Velocity: WT: Naive vs. Trained			0.229	ns
				Velocity: Null: Naive vs. Trained			0.318	ns
Lifespan correlation with different features	Fig. S13	Pearson bivariate correlations test	14	Lifespan vs. Two front paws Coordination index	r = 0.332		0.246	ns
		Pearson bivariate correlations test	9	Lifespan vs. Open arm entries	r = -0.253		0.512	ns
		Pearson bivariate correlations test	9	Lifespan vs. Latency to fall	r = -0.059		0.880	ns

Legends for Movies S1-S4

Movie S1. **Representative example of mouse running on computerized wheel.** Shown is a WT mouse running on the wheel set at speed 60 mm/sec. The video on the left was taken on Day 2; the video on the right was taken on Day 10. The most noticeable change is that early on, the WT mouse "skids" along the surface; by Day 10, it is accustomed to the rotation and changing speeds, and is able to better pace itself.

Movie S2. **Representative example of a Mecp2-null mouse running on computerized wheel.** Shown is a mutant mouse. Again, the biggest change from Day 2 (left) to Day 10 (right) is that the mouse is very tentative moving its limbs early on, but becomes more accustomed to the changing speeds and keeps up with the wheel by Day 10.

Movie S3. **A training-naïve Mecp2-null mouse on the ladder test.** The mice that were not trained on the computerized, changing-speed wheel have difficulty with the ladder test, moving slowly, lifting the tail for balance, occasionally missing rungs or stumbling.

Movie S4. **A trained Mecp2-null mouse performs like WT on the ladder test.** After training on the computerized wheel for 14 days, trained mutant mice moved easily and quickly across the ladder.
